# Method comparison of microscopy, metabarcoding, and multispectral imaging flow cytometry for identification and relative abundance analysis of insect-dispersed pollen

**DOI:** 10.1038/s41598-026-47800-3

**Published:** 2026-04-16

**Authors:** Elena Motivans Švara, Demetra Rakosy, Tiffany M. Knight, Alexander Keller, Thomas Hornick, Franziska Walther, Susanne Dunker

**Affiliations:** 1 Department of Species Interaction Ecology, Helmholtz Centre for Environmental Research – UFZ, Permoserstraße 15, 04318 Leipzig, Germany; 2 German Centre for Integrative Biodiversity Research Halle-Jena-Leipzig – iDiv e.V., Puschstraße 4, 04103 Leipzig, Germany; 3 Martin Luther University Halle-Wittenberg, Am Kirchtor 1, 06108 Halle (Saale), Germany; 4 Thünen Institute for Biodiversity, Bundesallee 65, 38116 Braunschweig, Germany; 5 Cellular and Organismic Networks, Biozentrum, Ludwig-Maximilians-Universität München, Großhaderner Straße 2-4, 82152 Martinsried, Germany; 6 Department of Physiological Diversity, Helmholtz Centre for Environmental Research – UFZ, Permoserstraße 15, 04318 Leipzig, Germany

**Keywords:** Pollen identification, Relative abundance, High-throughput, Artificial mixtures, Zoophilous pollen, Biological techniques, Ecology, Ecology, Plant sciences

## Abstract

Pollen identification and quantification are essential in ecological and evolutionary research to address plant-pollinator relationships, pollination services, and plant reproduction. Research into pollen transfer patterns has been mainly based on traditional light microscopy, however, it is time consuming, labour intensive, and requires taxonomic expertise. High-throughput methods allow automated pollen identification across large temporal and spatial scales. This study compares the accuracy of species identification and quantification of their relative pollen abundance using traditional microscopy, and two high-throughput methods, namely multispectral imaging flow cytometry (MIFC) and metabarcoding. Method performance was tested using artificial samples with known pollen species composition and pollen samples from pollinators with unknown pollen composition. After checking the agreement against the line of identity, the coefficient of determination (R^2^) values of linear models between the method estimates were compared. Metabarcoding performed best at identifying the taxa from artificial mixtures, while the two other methods assessed the relative abundance most accurately when there was information about species identity. Comparability between methods was overall low when assessing pollen composition on pollinators. To acquire both pollen identity and relative abundance, we recommend a metabarcoding-guided MIFC analysis that can be used as a high throughput approach for pollen research at large spatial and temporal scales.

**Supplementary Information:**

The online version contains supplementary material available at 10.1038/s41598-026-47800-3.

## Introduction

Most wild plants and crops rely on animal pollinators to deliver conspecific pollen in order to reproduce^1,2^, and many pollinators and other animals synergistically benefit from pollen nutrients^3^. Therefore, the identification and assessment of the relative abundance of animal-dispersed pollen is crucial for research aimed at understanding pollination and animal health^4^. Ecologists identify and quantify pollen on the bodies of insects or on the stigmas of plants to understand how the structure of plant-pollinator interactions changes across space and time^5–7^, determine relationships between pollen delivery and plant reproduction^8–10^, quantify the services provided by specific pollinator species^11–13^, assess the foraging breadth and nutritional intake of pollen by animals^14,15^, and evaluate the success of biodiversity measures such as flower strips^16^. Traditionally, pollen has been identified and quantified by light microscopy, however, new high-throughput methodologies for pollen identification based on microscopy in combination with machine learning as well as amplicon DNA sequencing demonstrate faster processing speeds than microscopy^17–19^ unlocking the potential for more comprehensive and larger scale pollination studies. To apply the most appropriate method given a project’s goals and resources, it is first necessary to compare the methods’ performance to each other, i.e., how precise and how fast they can identify species and measure relative abundance of pollen within a sample^20–22^.

Microscopy-based identification involves the magnification of pollen and is based on characteristics such as shape, size, and surface features^23^. The traditional method of light microscopy has been used for decades, and there are many publicly available online databases of pollen images^24,25^ and region-specific identification guides^26–28^. This method requires relatively basic equipment (e.g. a light microscope and slide preparation materials) but extensive taxonomic expertise^20,21^. The identification of pollen taxa is challenging as some pollen, especially those in the same genus or family, are morphologically similar, such as pollen in the Asteraceae family^29,30^, and yet others can strongly diverge despite being closely related, such as the *Myrsine* genus in the Primulaceae family^31^. Additionally, due to the long processing time, it is usually only possible to identify a subset of pollen grains in a sample (range from 50 to 200 grains minimum^21,26,32^). Therefore, rare taxa could be missed, resulting in an underestimation of richness^21^. Further, high pollen concentration or pollen clumping could result in the pollen grains overlapping on the slide surface, and covering the pollen grains under them, resulting in skewed estimates of relative abundance.

In contrast to traditional microscopy, the use of magnified pollen images in high-throughput machine learning approaches has substantially advanced automated pollen identification^17,19,33–36^. These methods have achieved high species identification accuracies (i.e. classification rates) between 85 and 98%^19,35,36^ and fast processing times. Recently, a method that uses multispectral imaging flow cytometry (MIFC) combined with a convolutional neural network (CNN) classifier that can consider morphological features of pollen as well as fluorescence and scatter properties to identify pollen grains has been developed^19,37^. The advantages of this method are its ability to capture additional information from pollen features and its rapid processing speed of up to 2000 particles per second, which can help to delineate morphologically similar taxa and allow for quick processing, respectively. The method yielded an accuracy of 93–96% at identifying pollen grains to species^19,37^and produced high accuracies even for plant species within the same family. However, the method requires specialised equipment and a reference database that captures the morphological variability of a population. As the pollen image reference library for multispectral imaging flow cytometry is in an early stage compared to other methods, absence of pollen taxa or their morphological variability in the library will influence the ability of this method to quantify richness and relative abundance^34,36–38^.

An alternative approach to morphology-based approaches is amplicon DNA-based pollen identification methods, which rely on DNA extraction, PCR amplification of one or more marker genes, and bioinformatic comparison with sequence databases. In the past decade, metabarcoding for pollen analysis has undergone optimization and testing^39–41^, and has been used for the identification of pollen species in mixed samples in numerous ecological studies investigating the foraging patterns of pollinators^42–48^. An advantage of metabarcoding is that it can make use of large established sequence reference libraries available for plants, such as NCBI GenBank, that contain a high coverage of plant species, particularly for well-studied regions such as Europe^49,50^. As metabarcoding uses differences in DNA base pairs rather than differences in morphology as a basis for the identification, it is also able to discriminate morphologically cryptic pollen species. Additionally, metabarcoding is better than microscopy at detecting rare plant species and yields higher estimates of species richness as just a small amount of DNA is necessary for species detection^20,47,51,52^. However, the exact methods used in metabarcoding can greatly differ from study to study^21^, e.g., the marker choice is particularly important, as it affects classification resolution, potential taxonomic amplification biases, database coverage, and DNA content (e.g. plastids versus nuclear)^21,40,51^. Additionally, metabarcoding may not perform as well or as consistently as other methods in assessing relative abundances of pollen taxa in a sample, as PCR-amplification can preferentially amplify certain taxa^53,54^. Copy number and DNA isolation can further contribute to quantification biases^55^.

These new high-throughput methods offer more efficient pollen identification and quantification, which would enable larger-scale research projects on zoophilous pollen than previously feasible. However, comparing the performance of light microscopy, MIFC, and metabarcoding in identifying pollen taxa and quantifying their relative abundance in mixed samples is essential. So far, comparisons using a known pollen mixture have only been conducted to compare light microscopy to metabarcoding. Metabarcoding showed high accuracy in identifying the species in a known pollen mixture^55–57^, with few false positives or negatives, but yielded only a weak positive correlation between the proportion of sequencing reads and the actual pollen relative abundance^20,51,55,56,58^.

Our study aims to compare all three methods with an artificial pollen mixture with known relative proportions to assess how each method performs at pollen identification and at quantifying pollen relative abundance. The performance of each of the three methods on the artificial mixture has implications on which method, or which combination of methods, might be most useful for ecologists researching unknown pollen mixtures, for example, to assess the identity and relative abundance of pollen taxa on the body of a pollinator. Taking this into consideration, the second part of our study aims at quantifying how well the methods agree on the identity and relative abundance of pollen present on the bodies of insects. This enables recommendations on method suitability, potentially including combinations like metabarcoding-guided analysis (e.g., metabarcoding for identification, microscopy/MIFC for quantification).

For identifying pollen taxa present in the artificial mixture, we hypothesise that (1) DNA-based metabarcoding performs better in identifying species than morphology-based identification methods (microscopy/MIFC) that may be limited in regard to pollen species with high intraspecific variability^38^ or cryptic morphological features. We also hypothesise that (2) MIFC will outperform traditional microscopy and metabarcoding in quantifying pollen relative abundance when species identity is known as it identifies a much larger number of individual pollen grains at high throughput. Finally, we hypothesise that (3) metabarcoding-guided MIFC analyses should compare best with quantitative assessments of metabarcoding-guided microscopy for unknown insect pollen samples.

## Methods

### Samples

Pure pollen samples for the artificial mixtures and pollen from insects caught in the field were taken from highly diverse, extensively-managed hay meadows in Transylvania, Romania. For the pure pollen samples used for the artificial mixtures, the florets of nine species of flowering herbs (Table 1) were identified in the field and collected in August 2020 (sampling location coordinates: 46.540217, 23.332937 WGS84). The plant species represent six families and five orders. The selection of individual species aimed at including common species that are attractive for pollinators, bloomed during our sampling time, and covered different taxonomic families and orders, thereby encompassing a range of pollen morphologies and sizes. Flower heads were dried and stored in separate vials according to collected plant species. Pollen from insects was collected in the following way: thirty pollinators (ten bumblebees, ten solitary bees, and ten flies) visiting flowers at the peak flowering time in the second half of July 2018 (locations were situated in the Apuseni Mountains, namely: Ocoale, 46.501057, 22.813802; Ghetari, 46.489428, 22.830505; Scarita-Belioara, 46.496185, 23.364144; and Rasca, 46.709965, 23.179110) were frozen in individual vials (to reduce the risk of pollen-cross contamination). The insects were removed and pinned, while the vials, which contained pollen fallen off from the insects, were stored at room temperature until further analysis. Pollen grains were extracted from the florets and insect vials by adding 1 mL and 1.5 mL, respectively, of pollen isolation buffer to sufficiently cover the surface containing pollen grains. The pollen isolation buffer (100 mM KH_2_PO_4_, pH 7.5; 1 mM EDTA; 0.1% (v/v) Triton X-100^19,59^) prevents pollen lysis by balancing the osmotic potential (KH_2_PO_4_), acting as a complexing agent to hinder aggregate formation (EDTA), and enabling pollen with hydrophobic surfaces to be better suspended (surfactant Triton). The pollen samples were then vortexed and sonicated for 5 min at room temperature in an ultrasonic bath to separate aggregated pollen grains. Each sample was filtered through a clean 100 µm filter into a 1.5 ml Eppendorf tube and centrifuged for 2 min at 4000 g. The supernatant was discarded and 70 µL Dulbecco’s phosphate buffered saline (without calcium, without magnesium) was added to the pellet.

**Table 1:** The characteristics of the pollen species selected for the artificial pollen mixtures.

Species	Family	Order	Pollen grain class	Pollen grain size (µm)
*Centaurea phrygia* L.	Asteraceae	Asterales	Medium	31.8–40.3 (36.4)
*Hypericum perforatum* L.	Hypericaceae	Malpighiales	Small	15.1–23.5 (18.7)
*Lathyrus pratensis* L.	Fabaceae	Fabales	Medium	38.3–44.8 (41.5)
*Leontodon hispidus* L.	Asteraceae	Asterales	Medium	39.5–49.5 (45.1)
*Lotus corniculatus* L.	Fabaceae	Fabales	Small	15.9–21.2 (19.4)
*Plantago lanceolata* L.	Plantaginaceae	Lamiales	Medium	22.3–27.8 (25.4)
*Potentilla erecta* (L.) Reausch.	Rosaceae	Rosales	Medium	25.5–34.5 (29.5)
*Prunella grandiflora* (L.) Scholler.	Lamiaceae	Lamiales	Medium	41.3–49.3 (45.2)
*Stachys officinalis* (L.) Trevis.	Lamiaceae	Lamiales	Medium	31.8–40.8 (36.1)

Pollen grain size information was taken from the “Leitfaden der Pollenbestimmung für Mitteleuropa und angrenzender Gebiete” (Beug, 2004), being based on the measurement of the pollen grains of 50 individuals of each species and at least two different pollen origins. The pollen grain size in the table is written as “*Minimum size of pollen*—*maximum size of pollen* (*mean*)”.

For the artificial mixtures, the pollen concentration was calculated based on the following procedure: for each plant species, the prepared pollen sample was vortexed and the number of pollen grains were counted in a Neubauer improved counting chamber. Four entire chambers were counted for all species except for *Hypericum perforatum* and *Stachys officinalis* where one to two thirds of each of the four chambers were counted due to high pollen concentration, and where three chambers were counted for *Centaurea phrygia* (means and standard errors in Supplementary Table S1). A minimum of 200 pollen grains was counted for each sample except for *Leontodon hispidus* where 93 pollen grains were counted due to low pollen sample concentration. Three different pollen master mixes (i.e. treatments) using the same nine flowering plant species were created (Table 2). A concurrent study in the same region (Rakosy, unpublished data) found that individual insects carried pollen from 0–9 species. Accordingly, nine species were selected to represent the most complex pollen mixture scenario. All master mixes used the same set of relative pollen proportions, which reflected pollen proportions found on insects from the same study (Rakosy, unpublished data). The three master mixes differed only in which species was assigned to each proportion. Namely, the abundance of two small grained (less than 20 µm large) pollen species (*Lotus corniculatus* and *Hypericum perforatum*) was manipulated in the mixtures (Table 2). Treatment SmallDom included the small-grained pollen at high relative abundance, treatment SmallInt included the small-grained pollen at intermediate relative abundance, and treatment SmallRar included the small-grained pollen at low relative abundance. This approach was chosen to be able to assess the influence of pollen size on pollen grain detection. The master mix was vortexed before aliquots were withdrawn, amounting to 10 aliquots in total from each master mix for each method. Two negative controls consisting of 70 µL Dulbecco’s phosphate buffered saline were run for each method. One positive control consisting of pure samples of each pollen species (~ 3000 pollen grains per control) was run per method. If a positive control for microscopy contained other species, the true proportion was adjusted accordingly, by calculating how many pollen grains of each species were put into the mixture (Supplementary Table S2).

**Table 2:** The set-up of the artificial pollen mixtures experiment for treatment SmallDom, treatment SmallInt, and treatment SmallRar.

Species	Proportion of species in mixture (%)	Number of aliquots per method	Number of pollen grains per aliquot
Treatment SmallDom			
*Leontodon hispidus*	1.254	10	5000
*Lathyrus pratensis*	1.254		
*Prunella grandiflora*	1.254		
*Stachys officinalis*	2.508		
*Centaurea phrygia*	5.015		
*Plantago lanceolata*	11.107		
*Potentilla erecta*	15.167		
***Lotus corniculatus***	25.929		
***Hypericum perforatum***	36.513		
Treatment SmallInt			
*Leontodon hispidus*	1.254	10	5000
*Lathyrus pratensis*	1.254		
*Prunella grandiflora*	1.254		
*Potentilla erecta*	2.508		
***Lotus corniculatus***	5.015		
***Hypericum perforatum***	11.107		
*Stachys officinalis*	15.167		
*Centaurea phrygia*	25.929		
*Plantago lanceolata*	36.513		
Treatment SmallRar			
***Lotus corniculatus***	1.254	10	5000
***Hypericum perforatum***	1.254		
*Potentilla erecta*	1.254		
*Leontodon hispidus*	2.508		
*Lathyrus pratensis*	5.015		
*Prunella grandiflora*	11.107		
*Stachys officinalis*	15.165		
*Centaurea phrygia*	25.929		
*Plantago lanceolata*	36.513		

“Small” pollen-grained species are highlighted in bold.

The pollen samples collected from individual insects contained the mixture of pollen species collected by that pollinator and each was therefore vortexed and split into equal parts for microscopy, metabarcoding, and MIFC.

### Establishment of MIFC reference database

As the image reference database was not previously established for MIFC, we established a database consisting of the pollen of 68 plant species collected by botanists in the study region. The pollen was isolated with the same protocol as for the artificial pollen mixtures. Pollen was measured with an imaging flow cytometer ImageStream® MK II (Amnis part of Cytek, Amsterdam, Netherlands) equipped with three lasers (488 m laser intensity 5 mW, 561 nm laser intensity 20 mW and 785 nm laser intensity 0.1 mW) and two cameras (Dunker et al. 2021). The instrument used has a special order configuration for pollen analysis (Patent submission PCT/EP2017/075553, WO 2019/068352 A 1). In brief, one brightfield image, 4–5 fluorescence images, and one scatter image were recorded simultaneously on each of the cameras with 40× magnification, a numeric aperture of 0.75, a pixel size of 0.5 × 0.5 µm, and a 60 × 128 µm field of view. Dulbecco’s phosphate-buffered saline without calcium and magnesium was used as sheath-fluid. For each sample, the image acquisition concluded when either 5,000 particles were measured or a maximum time of 10 min had elapsed. Images were collected with the instrument-specific INSPIRE Software (Version 200.1.620.1) and processed with the IDEAS Software (Version 6.2.187.0). The derived pollen images were filtered by removing blurred images and images with debris or without pollen by using bivariate plots of brightfield image intensities and mean background intensity according to Hornick et al. (2025). For machine learning, a manual inspection of all remaining images was performed in the IDEAS Software to guarantee a high qualitative training dataset of pollen images representing single classes for training a data-set-specific convolutional neural network. Thereby, remaining particles other than pollen, as well as heterospecific pollen species, were removed, resulting in 82,238 pollen images for the training dataset.

### Metabarcoding

The pollen samples for metabarcoding were dry-homogenised (TissueLyser, Qiagen, Venlo, Netherlands) with 3 mm tungsten carbide beads for 1.5 min and DNA was extracted with the NucleoSpin Food kit (Macherey–Nagel, Düren, Germany) following the “isolation of genomic DNA from honey or pollen” supplementary protocol. A dual-index pooled amplicon library on the ITS2 genomic region was produced according to Sickel et al. (2015). Sequencing was performed on an Illumina MiSeq with 2 × 300 bp by the company AIM—Advanced Identification Methods GmbH (Leipzig, Germany). Bioinformatics followed the procedure described in Campos et al. (2021) with a few modifications: VSEARCH v2.14.2^60^ was first used to join paired ends of forward and reverse reads, but only the forward reads were used as they covered the entire ITS2 fragment length and the quality of the reverse reads was insufficient. Consequently, VSEARCH was used for quality end-trimming of forward reads to reach maximum expected errors (max EE < 1^61^) over the entire read. Reads shorter than 200 base pairs were removed and de-novo chimera filtering was performed^62^. Finally, amplicon sequence variants (ASVs) were defined by denoising the data, as previously done successfully for pollen^48,63^. For all of the artificial mixture ASVs, global alignments were used against a global ITS2 reference database^64,65^. For reads that remained unclassified, SINTAX^66^ was used to assign taxonomic levels as deeply as possible, while maintaining a maximum of genus level with the same global reference database. For the insect pollen sample ASVs, global alignments and an identity cut-off threshold of 97% against a plant ITS2 reference database generated with the BCdatabaser^67^, which consisted of plants recorded for the study region, were used. For the remaining unclassified ASVs of insect samples, the same protocol was used as for the artificial mixture samples.

### Multispectral imaging flow cytometry

The pollen mixtures were imaged with an imaging flow cytometer with the specifications detailed above regarding the establishment of the MIFC reference database. A convolutional neural network (CNN) model was created with the IDEAS® 6.3 and Amnis® AI software (Amnis part of Cytek, Amsterdam, Netherlands) including either 35 classes (genera level of all collected 68 plant species) or the 9 reference classes (genera) for the “informed” training and the prediction of the artificial mixtures. The software uses a TensorFlow version 1.7.0 library and the CNN model architecture VGG16 network^68^. For the model, two brightfield image channels (Ch01, Ch09), the scatter channel (Ch06), and four fluorescence channels (Ch02-Ch05) were used with a maximum image size of 150 pixels.

### Microscopy

Pollen mixtures for microscopic assessment were first combined with safranin gel^69^ to dye the pollen grains. The mixtures were mounted on microscopy slides and covered with a square cover slip. The light microscope was used at 50× magnification. A grid coordinate system was set up to facilitate the detection, counting, and image acquisition of pollen on the slides. A total of 100 coordinate points along the grid were checked for the artificial pollen mixtures (at 3 mm x-axis and 1 mm y-axis intervals). The number of coordinates checked for the insect pollen samples consisted of 50 points (at 3 mm x-axis and 2 mm y-axis intervals). If fewer than 30 pollen grains were identified using the grid coordinate system, all of the pollen grains on the slide were identified. At each coordinate where at least one pollen grain was observed, an image was taken with a DSLR camera attached to the microscope using a C-mount tube system. The coordinates of every image were recorded and in case an identification was not possible with the respective image, the taxonomist was able to re-check the respective slide and position. The pollen grains were later measured using ImageJ (Version 1.53e, National Institutes of Health, USA) with an objective micrometer (Th. Geyer GmbH & Co. KG, Renningen, Germany) to calibrate the images. Pollen grains were identified by a specialist using two online pollen libraries^25,28^ and one internal pollen library (Species Interaction Ecology Department, Helmholtz Centre for Environmental Research, Leipzig, Germany), as well as the Guide to the Pollen Analysis for Central Europe and the Adjacent Areas^26^.

### Data preparation

All identifications were performed at the genus and family level. Before analysis, plant taxa in a sample with a pollen grain proportion less than 1% abundance were excluded for all methods. To ensure that enough pollen grains were identified with each method to sufficiently capture the diversity of each sample, we calculated sample coverage (i.e. the proportion that the sampled genera comprise of the total individuals in the community) and sample coverage. We used the ‘iNEXT’ function in the iNEXT R package^70,71^ to generate coverage-based rarefaction curves for each method, reporting observed coverage in out$DataInfo$SC.

### Method comparison

#### Statistical analyses

*Artificial mixtures*: As a first step to assess which method was the most accurate at identifying the pollen taxa present in the artificial mixtures, we conducted a Kruskal–Wallis rank sum test with a pairwise Wilcoxon “Holm”-adjusted post-hoc test^72^ with the multcompView R package^73^. The data tested was the proportion of taxa (that were put into the mixture) detected for each treatment compared at the genus level (n = 30 for each treatment).

**Fig. 1:**
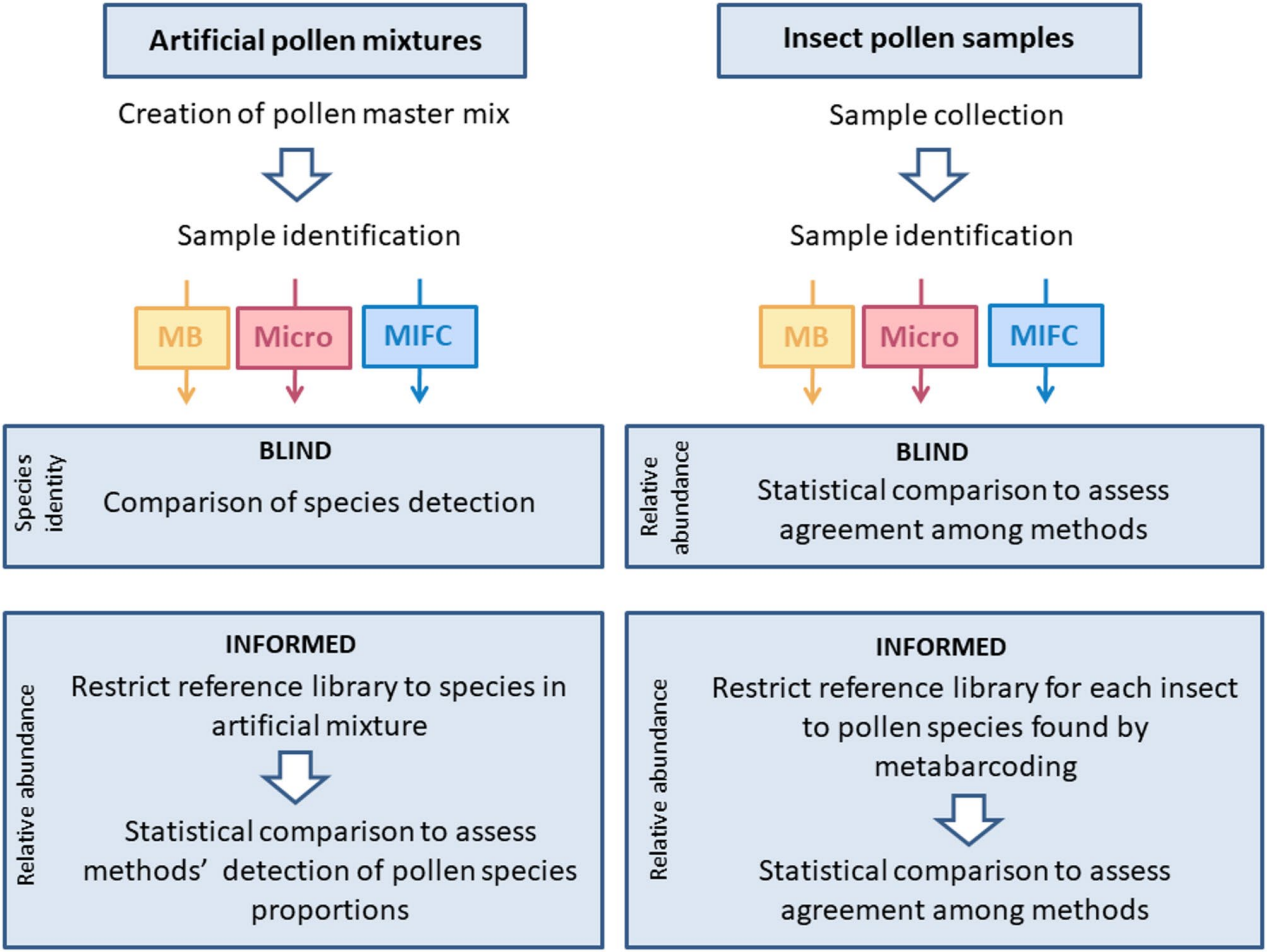
Diagram of the steps involved in preparing and analysing the pollen samples for the artificial mixtures and insect pollen (MB = metabarcoding, Micro = microscopy, MIFC = multispectral imaging flow cytometry).

In a second step, we evaluated which method performs best in determining quantitative results (i.e. pollen proportions in the mixtures). Two approaches were applied (Fig. 1): the “blind” analysis considered the raw identification results; if a pollen taxon was misidentified by a method or failed to be identified by a method, the relative abundance results would also suffer and not be comparable among methods. The second “informed” (knowledge of known composition) analysis corrected misidentifications and limited the reference libraries of all methods to those taxa present in the artificial sample. The “informed” analysis is thus aimed at comparing the abilities of the three methods to accurately quantify the relative proportion of pollen from a known pollen pool. A Kruskal–Wallis rank sum test and a subsequent pairwise Wilcoxon “Holm”-adjusted post-hoc test was applied to describe the significance levels of the relative proportions of each species in each mixture between the three methods. In addition, linear regressions were visualized with ggplot2 to compare the relationships between true proportions and proportions assessed by each method across the three treatments.

*Insect pollen samples*: For each of the thirty insect pollen samples, we assessed the agreement among methods using the same “blind” and “informed” approaches as described above. As metabarcoding was found to consistently have the highest taxa detection rate with the lowest number of false positives with the artificial mixtures, its results were used to define the reference libraries of microscopy and MIFC in the “informed” approach (i.e. based on metabarcoding results). To assess which methods were the closest at assessing taxon identity and relative abundance in the insect samples, linear regressions were visualized with ggplot2 to compare the relationships between proportions assessed by pairs of methods (i.e. metabarcoding-microscopy, metabarcoding-MIFC, and MIFC-microscopy).

We used R 4.4.1^74^ and the packages dplyr 1.1.4^75^, tidyr^76^, and ggplot2 3.5.1^77^ for data analysis and figure preparation.

## Results

### Artificial mixtures

In total, 90 pollen samples were analysed (10 aliquots per method per treatment). Multispectral imaging flow cytometry revealed 21,809 pollen grains, microscopy identified 1,581 pollen grains, and metabarcoding detected 1,296,820 reads (see Table 3 for means and standard deviation per method and treatment), excluding controls. The total number of plant genera found in the “blind” analysis with each method was the following: 21 plant genera were found with MIFC, 15 plant genera with microscopy, and 12 with metabarcoding, indicating false positives for each method.

**Table 3:** Mean and standard deviation (SD) of pollen grains and/or read abundance of ASVs identified in each treatment and method (MIFC-multispectral imaging flow cytometry) within A) the artificial mixture and B) each method of the insect pollen.

Method	Treatment	Mean	SD
(A) Artificial mixture			
MIFC	SmallDom	904.0	109.3
MIFC	SmallInt	627.9	70.5
MIFC	SmallRar	649.0	82.5
Microscopy	SmallDom	51.2	8.6
Microscopy	SmallInt	57.4	14.7
Microscopy	SmallRar	49.5	13.7
Metabarcoding	SmallDom	36507.6	7034.5
Metabarcoding	SmallInt	60635.6	20498.6
Metabarcoding	SmallRar	32538.8	5746.8
(B) Insect pollen			
MIFC		1140.8	589.7
Microscopy		104.2	158.7
Metabarcoding		2812.4	1953.9

The rarefaction results showed high observed sample coverage for each method (Supplementary Table S3 & S4), demonstrating that a sufficient number of pollen grains/reads were identified to capture the diversity in each sample. Sample coverage (ranging from 0 to 1, where values close to 1 indicate that nearly all individuals belong to detected species, with only extremely rare species remaining undetected) was 1.0 (standard deviation (SD) 0.0) for MIFC, 0.955 (SD 0.035) for microscopy, and 1.0 (SD 0.0) for metabarcoding for the artificial mixtures.

### Genera identification in artificial mixtures

**Fig. 2:**
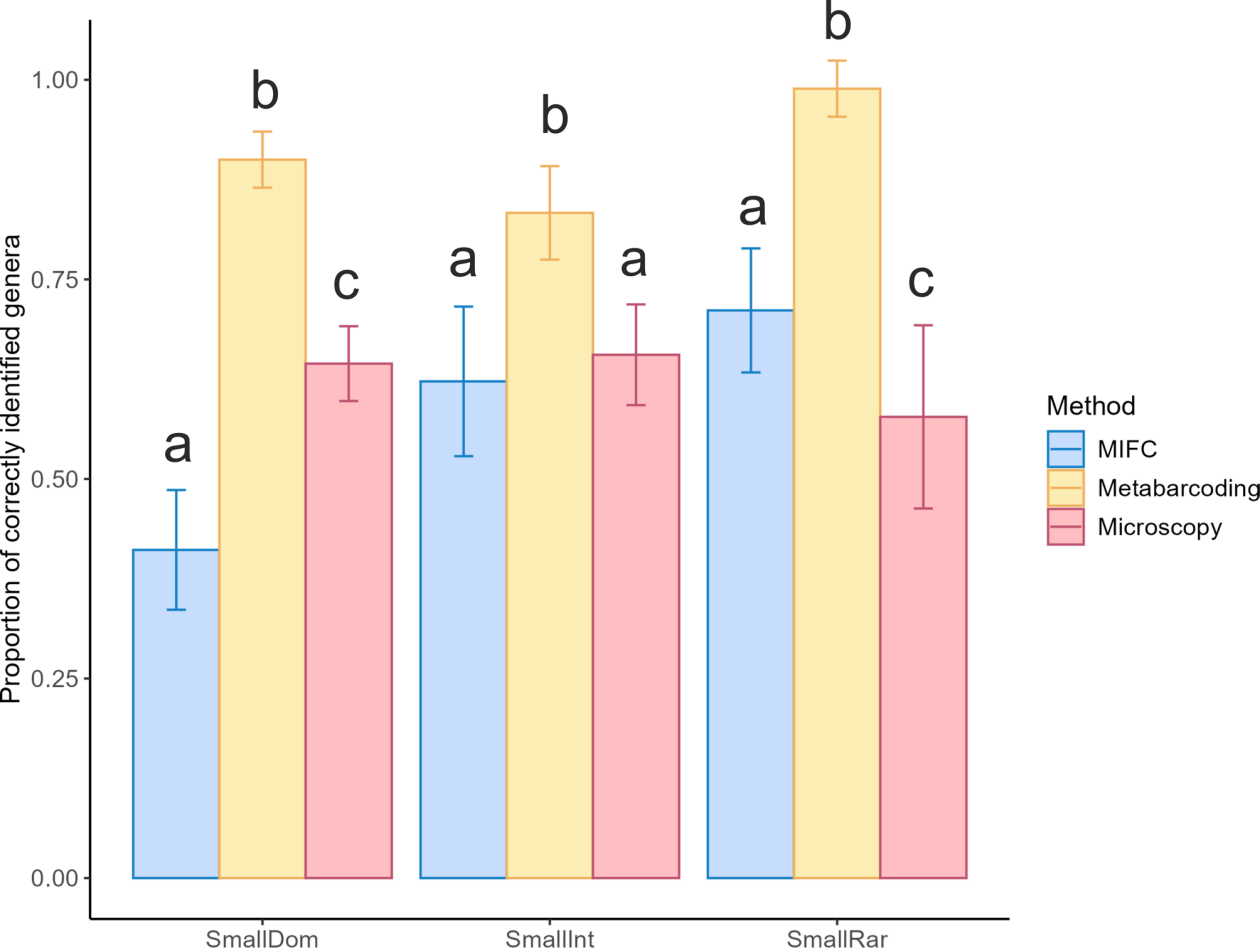
Mean ± 1 SD proportion of correctly identified genera (out of 9 in total) per method across treatments for the “blind” approach. A Holm-adjusted post-hoc test was performed to test for significant differences between the different methods quantifying pollen grains of one genus in the mixture (indicated by letters **a**, **b**, and **c**). Same letters denote no significant difference, whereas different letters indicate significant differences.

With the “blind” approach (i.e. taxa were identified with no prior knowledge of which taxa were in the mixture) (Hypothesis 1), metabarcoding identified a significantly higher proportion of correct genera in the mixtures than microscopy and MIFC in all treatments (Fig. 2, Supplementary Table S5 & S6). In treatment SmallDom, MIFC identified the lowest proportion of correctly predicted genera. For microscopy, *Lathyrus* and *Leontondon* were not detected in any treatments. For MIFC, *Lotus* and *Plantago* were not detected in any treatments, while *Centaurea* was not detected in treatment SmallDom. Metabarcoding did not detect *Prunella* in treatment SmallInt. After the “informed” identification, MIFC and microscopy identified all of the species in the artificial mixture.

### Relative abundance in artificial mixtures

**Fig. 3:**
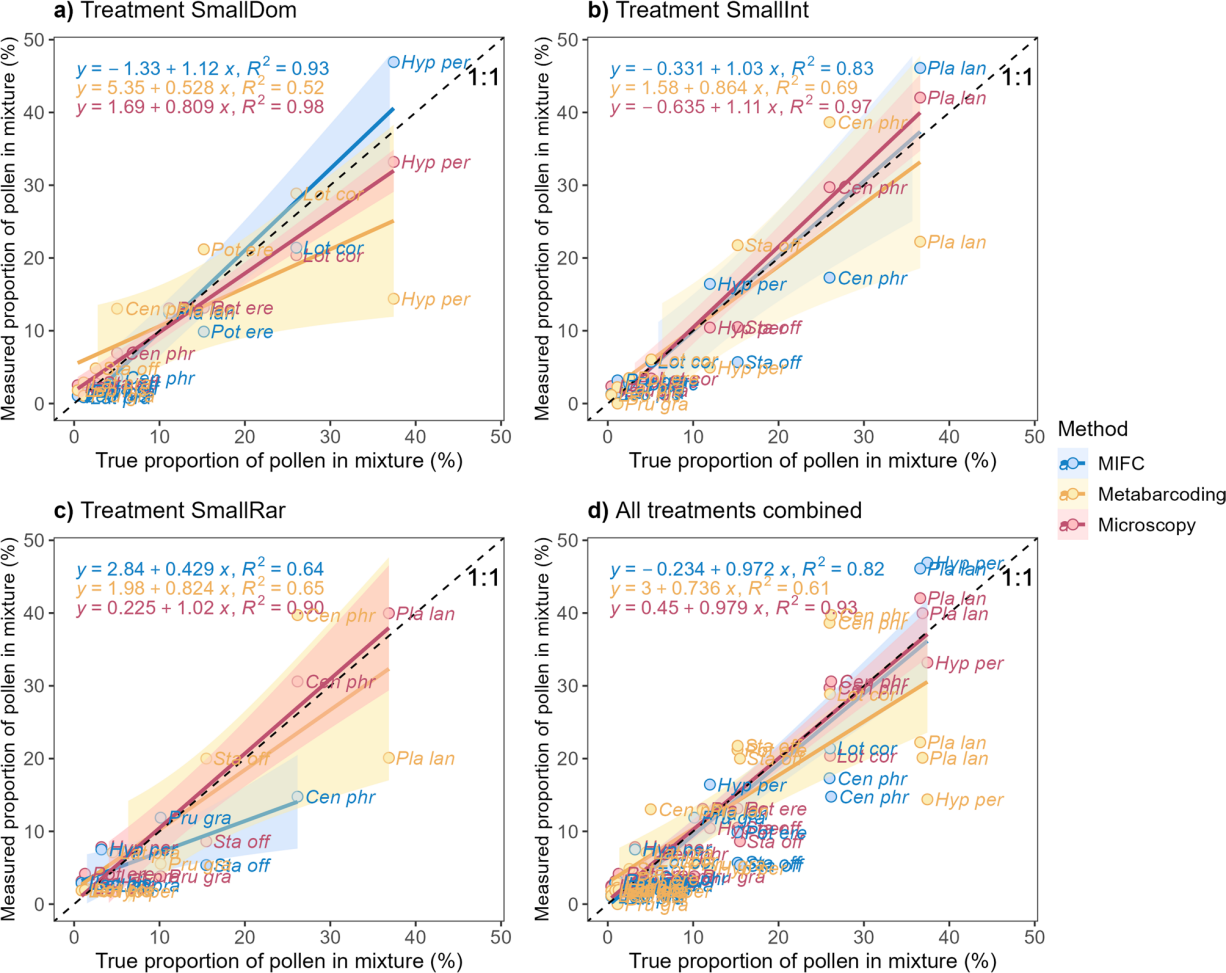
Linear regression lines show the variability of the identification results of each method explained by the true proportions (1:1 line) for the “informed” analysis for (**a**) treatment SmallDom, (**b**) treatment SmallInt, (**c**) treatment SmallRar, and (**d**) all treatments combined.

When all treatments were combined, the highest coefficient of determination, closely aligning the line of identity, were found for the true proportion-microscopy comparison (R^2^ = 0.93), followed by true proportion-MIFC (R^2^ = 0.82) and true proportion-metabarcoding (R^2^ = 0.61) (Fig. 3d). This trend in coefficient of determination ranking was consistent across individual treatments, except for treatment SmallRar where the true proportion-microscopy comparison (R^2^ = 0.90) outperformed true proportion-metabarcoding (R^2^ = 0.65) and true proportion-MIFC (R^2^ = 0.64) (Fig. 3c). The “blind” results showed similar results for microscopy and metabarcoding, however, the R^2^ was lower for MIFC (SmallDom: 0.03, SmallInt: 0.22, SmallRar: 0.27, Supplementary Figure S7).

**Fig. 4:**
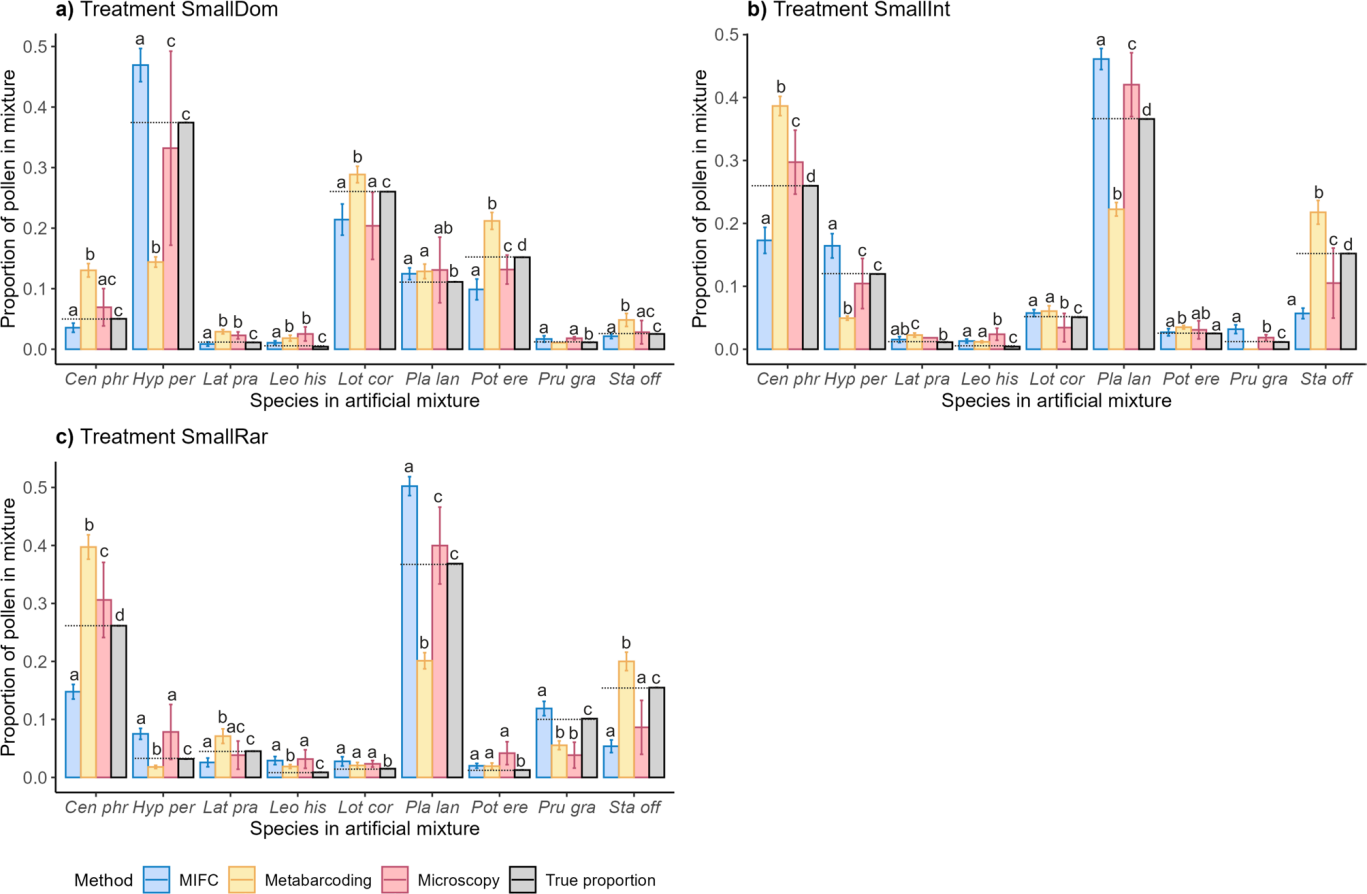
Mean proportion ± 1SD of each species for the three different methods in the artificial mixture treatments (**a**–**c**) (“informed” analysis). A pairwise Wilcoxon “Holm”-adjusted post-hoc test was performed to test for significant differences between the different methods quantifying pollen grains of each species in the mixture (indicated by letters a, b, c and d in figure). Species are abbreviated as the first three letters of the genus followed by the first three letters of the species epithet.

When comparing the relative proportions of the species in each treatment (Fig. 4, Supplementary Tables S8 & S9), microscopy consistently exhibited higher variation between the 10 technical replicates than both other methods. Metabarcoding consistently overestimated *Centaurea*, *Lathyrus*, *Leontondon*, *Stachys*, and underestimated *Hypericum* relative abundance in treatments SmallDom and SmallInt. In treatment SmallRar, having the lowest coefficient of determination, MIFC underestimated *Centaurea*, *Lathyrus*, *Stachys*, and overestimated *Hypericum*, *Leontondon*, *Lotus*, *Plantago*, and *Prunella*.

### Pollen samples from insects

In total, MIFC revealed 34,224 pollen grains, microscopy identified 3,126 pollen grains, and metabarcoding identified 84,371 reads (see Table 3 summarizing means ± 1 SD per method). In the “blind” identification, 38 pollen genera were found with microscopy, 31 plant genera with MIFC, and 39 with metabarcoding (Supplementary Figure S10). Sample coverage was 1.0 (SD 0.0) for MIFC, 0.992 (SD 0.019) for microscopy, and 1.0 (SD 0.0) for metabarcoding (Supplementary Table S3 & S4).

**Fig. 5:**
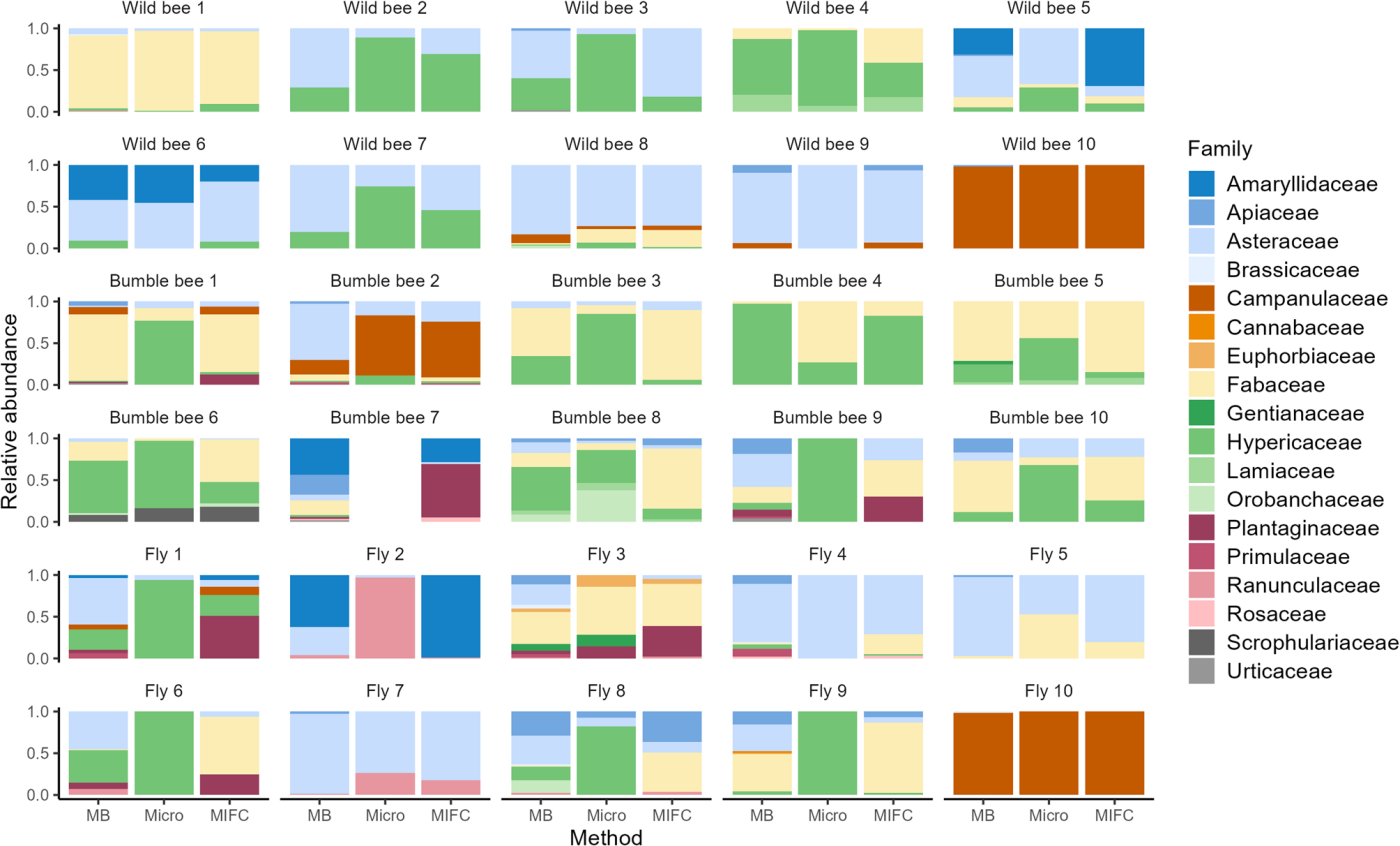
Relative proportions of pollen plant families among the three methods (Micro—microscopy, MIFC and MB—metabarcoding) estimated for pollen sampled from 30 pollinators that were collected in the field and analysed with the “informed” approach.

As the “informed” (metabarcoding-guided) morphological analyses represented the true proportions best in the artificial mixtures, we continued this approach for the insect pollen samples (30 pollen samples in total, collected in the field from 10 wild bees, 10 bumble bees, and 10 flies). In the “informed” identification, restricted to the 39 genera identified with metabarcoding, 25 plant genera were identified with microscopy and 31 plant genera with MIFC. Via microscopy, generally fewer pollen classes were determined, while metabarcoding and subsequently MIFC predicted a higher number of pollen classes. For some samples with one dominating pollen class, all methods revealed comparable relative abundances (e.g., wild bee 9, wild bee 10, and fly 10) (Fig. 5). Although overall comparability between methods was low (Supplementary Figure S11 & S12), surprisingly, the two morphological methods (microscopy and MIFC) exhibited the weakest agreement (R^2^ = 0.06), while both displayed higher correspondence with metabarcoding (microscopy–metabarcoding: R^2^ = 0.24; MIFC–metabarcoding: R^2^ = 0.26).

### Controls

Pollen for the positive controls was collected directly from flowers in the field, so a certain level of contamination with heterospecific pollen cannot be excluded. Six of the positive controls used for microscopy contained only the target species. The detected contamination rates for each method can be found in Supplementary Table S13.

In microscopy and MIFC negative controls, no pollen was detected for both the artificial mixture and the insect pollen samples. In the metabarcoding artificial mixture negative controls, 11 taxa were found in total, with two target taxa (*Centaurea* and *Plantago*). In the metabarcoding insect pollen negative controls, 23 taxa were found with fewer than 65 reads (Supplementary Table S13). *Acer pseudoplatanus*, *Crepis biennis*, and *Hedera helix* each occurred with more than 100 reads in the negative controls (Supplementary Table S13). To minimise contamination effects, we excluded observations of these three species (4 observations across three samples) from the metabarcoding data when read counts were lower than those observed in the negative controls.

## Discussion

We compared three different methods for the identification and relative abundance assessment of insect-dispersed pollen. As expected, metabarcoding performed best at identifying the pollen taxa in the artificial mixture samples without prior knowledge of the species composition, while microscopy-based methods (i.e. light microscopy and MIFC) were generally more accurate than metabarcoding at quantifying the relative abundance of pollen taxa when species identity is known. However, microscopy resulted in the highest agreement with the 1:1 line for relative abundance quantification, in contrast to our hypothesis that MIFC would be the most accurate. When comparing the assessment of insect pollen samples, we find little agreement among methods, in contrast to our hypothesis that MIFC and microscopy would have a stronger concordance in their estimation of relative pollen taxa abundance.

### Factors influencing the identification of pollen taxa in artificial mixtures

Pollen identification can be affected by multiple factors^55,78^. In the present study, three key factors determining the accuracy of the method were: (1) the ability to distinguish interspecific morphological variation, (2) the ability to distinguish intraspecific morphological variation^30,38,79^, and (3) the storage conditions of the pollen samples. There are many genera that are closely related and very difficult to delineate morphologically due to many of the same diagnostic characteristics (e.g. size class, pollen class, polarity, shape, aperture number, aperture condition)^25,26^. Additionally, intraspecific pollen morphology variation is common in angiosperms, with one third of families possessing heteromorphic taxa^80^. Specifically, the shape of pollen grains, sculptures, aperture types and numbers, and structure are known to vary within species and have been associated with floral and sexual diversity^81^. In addition, storage conditions can also play a major role for the identification of pollen, as e.g., pollen size and morphology is affected by different storage treatments^82^, though it has been shown to not influence metabarcoding results^83^.

In particular, the two morphology-based methods, light microscopy and MIFC, were influenced by these three factors. Light microscopy counts fewer pollen grains than the other methods and is known to have difficulty distinguishing morphologically similar pollen taxa^30^. Indeed, in our light microscopy identification, the following morphologically similar taxa were often difficult to distinguish: *Prunella* and *Thymus*, *Leontodon* and *Crepis*, and *Lathyrus* and *Vicia*. MIFC has the advantage of using a more diverse set of parameters (e.g. related to shape, size, and fluorescence) to detect subtle differences that would not be possible to ascertain with the human eye. However, a challenge for automated pollen recognition from MIFC images using neural networks is identifying grains that differ from images in the reference database either by intraspecific morphological variation of size, shape and fluorescence^38^ or by storage conditions. In our case, pollen grains that were dehydrated in contrast to reference data resulted in several misclassifications (e.g. for *Lotus* and *Hypericum*) (Supplementary Table S14). Other factors that influence pollen grain size and shape (e.g. geographic variation^79^), pollen developmental stage, presence of degraded pollen, and ploidy level^38,59^ likely influenced the “blind” identification of pollen mixtures, although high accuracies have still been achieved in previous studies^19,37^. It is known that these models can fail if the testing conditions are more challenging^36,84^ and the model does not assign pollen grains with a slightly deviating morphology correctly but instead to classes with more similar properties. Finally, the choice of the nine plant species in the artificial mixture may have also affected the outcome of the morphological methods, as the selection influenced the degree of differentiation between the species, as well as the proportion of morphologically cryptic pollen species. However, the selection was aimed to allow the testing of the three methods under realistic field conditions, and identification is expected to be comparable with other pollen species sets.

Metabarcoding was not dependent on morphology for the identification and was therefore able to most consistently and accurately identify the pollen taxa in the artificial pollen mixture with the least false positives and negatives. This accuracy in identification has been shown before for pollen species^55,85,86^. This high accuracy is likely because DNA methods can rely on extensive established databases (66–89% of known plant species are covered by genetic databases in Europe for the ITS2 marker as used here^50^), better distinguish morphologically similar (or even cryptic) pollen taxa, and recognize pollen at different developmental stages^87^. Indeed, metabarcoding was the only method that was able to successfully resolve the taxa to the species, rather than the genus, level.

### Factors influencing relative abundance assessment in artificial mixtures

In the “blind” analysis, microscopic assessment was able to accurately estimate the relative abundance of pollen, though misidentification still occurred for morphologically similar species. Notably, however, the relative abundance of the most prevalent taxa—such as Plantago and Hypericum, depending upon the experimental treatment—were consistently identified with high accuracy. On the other hand, MIFC performed weakly in the “blind” analyses; a possible reason is the morphological differences between the reference images and samples affecting relative abundance as well as identification.

Once the methods were corrected for the identity of taxa in the sample (“informed” analysis), relative abundance of those taxa was best assessed by light microscopy. Microscopy was the method providing the closest match to the true proportion of pollen taxa in the mixture, though the variation was higher than for the other methods. This high variation could be due to the subjectivity of the observer^88^ or the smaller sample size of counted pollen. Although MIFC was not the most accurate method as hypothesized, it still achieved generally high R^2^ values and greater precision. When time requirement for measurements is taken into account, the so far slightly lower performance of MIFC for relative abundance quantification might be acceptable. As expected based on previous studies, metabarcoding performed worse at estimating relative abundance, because the DNA content/copy number extracted and measured by this method is not entirely correlated to the pollen grain number^21^. A closer look at the individual pollen genera in the mixtures revealed that the same genera, namely *Hypericum*, *Centaurea*, *Lathyrus*, *Leontondon*, and *Stachys* were consistently under- or over-represented in the sequencing analyses. The same pattern was found in other previous studies^51,55^. Explanations could be variability marker gene copy number per grain, as well as ploidy level^87^.

In general, the slightly lower R^2^ for true proportion versus MIFC was due to an overestimation for *Hypericum* and *Plantago* as well as underestimation for *Centaurea* and *Stachys* for MIFC in treatment SmallInt and SmallRar. The generally lower R^2^ values for relative proportions (true vs. metabarcoding) can be largely attributed to the compositional dependency of proportions, with strong underestimations of *Hypericum* and *Plantago,* the two taxa most poorly estimated across all mixtures. Changes in morphology due to different storage conditions compared to pollen in the image reference database may influence pollen identification as the overestimation of *Hypericum* by MIFC in treatment SmallRar and the underestimation of *Hypericum* by metabarcoding across treatments contributed to their low fit. However, further testing is required to elucidate the effect on pollen size on detection and relative abundance assessment.

### Method comparison of insect pollen identification

Given the results for the artificial mixtures, we expected that estimates of the relative abundance of pollen taxa on insects would be similar for the microscopy- and MIFC-based methods and that these would differ from metabarcoding estimates (once the identity of the pollen taxa was corrected based on metabarcoding; i.e., the “informed” analysis). However, we found that there was little comparability among methods. Single-taxon dominated samples were correctly identified by all three methods (e.g. fly 10, wild bee 10), while the other samples contained different taxa or starkly contrasting proportions of the most common taxa. It is not possible to say whether the differences stem from compositional effects or correct determination of species identity as the true proportion could not be determined as in the artificial mixtures. However, the following general trends were observed: (1) Microscopy identified fewer grains and often fewer taxa than the two high throughput methods. (2) Light microscopy and MIFC both had difficulty distinguishing between *Hypericum* and *Lotus* (e.g., bumble bee 3, bumble bee 4, bumble bee 5) due to dehydrated pollen grains in the samples. Although measures were taken to prevent clumping during sample preparation^19,59^, pollen clumping during the creation of the aliquots, causing compositional differences, cannot be fully excluded.

Using metabarcoding to guide microscopy and MIFC (i.e., by providing species identifications) influences the outcomes of these morphological methods. For example, it can introduce false negatives, as microscopy or MIFC may detect taxa absent from the metabarcoding species list due to primer mismatches, low DNA yield, or sample differences^89,90^. Despite these limitations, metabarcoding remains one of the most powerful and widely adopted methods for species identification, particularly in biodiversity monitoring programs^91,92^. Thus, when dealing with field-collected samples of unknown composition—as is typical in most ecological studies—it remains the only practical option for initial species identification.

### Current state and future perspectives for each method

As a nascent method with a reference library created for our study system, MIFC assessed relative abundance well and has the potential to accurately identify taxa “blindly”. To accurately identify pollen, high throughput microscopy-based solutions need to account for the morphological variability of pollen in real world samples^38^. One approach is to establish pollen reference libraries that comprehensively cover morphological variation by taking multiple pollen samples to cover temporal and spatial variability^36^ as well as the species present in the relevant region. Sufficiently covering variation can help to prevent the problem of short-cut learning and overfitting of machine learning approaches, learning the specific patterns present in the training data to an excessive degree, thereby enabling it to perform very well on the training data but failing to generalize to new, unseen data^36,84,93^. Other approaches, such as unsupervised cluster approaches of MIFC data^37^, have been developed and will be important to expand upon further in the future.

Metabarcoding excelled at species identification, but should be interpreted with care in terms of relative abundance results. Specifically, studies that use pollen metabarcoding should apply appropriate analytical methods, such as rank-based statistics and non-linear models^15,87^ to assess species abundance or categorically describe detected species’ abundances as, for instance, “abundant” and “rare” occurrences based on a threshold. At the moment, we cannot clearly elaborate whether the abundance discrepancies are systematic (e.g. gene copy numbers, genome size, grain rigidity or taxonomy) or random (e.g. PCR) or a combination of both^87^. The development of analytical methods^94^ that allow for correction might improve metabarcoding quantification for systematic biases and PCR-independent approaches (e.g. metagenomics)^57^ might improve random quantification biases in the future.

Although light microscopy showed some limitations in accurately distinguishing among morphologically similar species, it nonetheless performed well in quantifying relative pollen abundance, demonstrating that it remains a valuable and reliable method for pollen analysis. Some of the method’s limitations can stem from improper sample preparation, biased image acquisition (i.e. missing pollen grains that are at different levels on the slide), and the level of taxonomic expertise^37^. A recommendation for the future of light microscopy for pollen identification is to use and publish clearly described protocols^95^ for sample preparation, pollen grain counting, and identification for cross-study comparability^96^.

### Recommendations for pollination researchers

The selection of a pollen assessment method depends on the information required for the study (i.e. identity, relative abundance) and other factors. Based on our results, we provide a table of recommendations for different method(s) depending on the study (Table 4). In summary, we recommend metabarcoding to identify pollen as it is the most accurate method. When only relative abundance is required, either light microscopy or MIFC can be selected as both performed well in terms of assessing relative abundance in the artificial mixtures. We recommend MIFC for higher numbers of samples and taxa, as well as multiple samplings as it would provide a time advantage to light microscopy in these cases. When using CNN-based automated pollen identification from MIFC images, we recommend storing the reference pollen samples the same way as the samples that should finally be predicted.

**Table 4:** Recommendations for the selection of a pollen assessment method based on the information of interest and the study characteristics based on our results.

		Information needed		
		Identity	Relative abundance	Identity and relative abundance
Sample size	Small (< 10 samples)	MB	Micro	MB + Micro
	Large (> 10 samples)	MB	MIFC	MB + MIFC
Floral diversity	Low (< 5 species)	MB	Micro	MB + Micro
	High (5 + species)	MB	MIFC	MB + MIFC
Analysis time	Analysis time high (e.g. few samplings)	MB	Micro	MB + Micro
	Analysis time low (e.g. Multiple samplings)	MB	MIFC	MB + MIFC
Financial resources	Limiting	MB/Micro	Micro	MB + Micro
	Non-limiting	MB	MIFC	MB + MIFC

However, when both identification and relative abundance information is required, no single method is sufficient. Therefore, we recommend a two-step process in which pollen taxa are first identified by metabarcoding and then these data are used to inform a relative abundance assessment by light microscopy or MIFC. A combination of metabarcoding and microscopy has already been suggested for pollen^39^ and other study systems such as microalgae^97^ and microbial eukaryotes^98^ to provide valuable information about composition. Other pollination studies have noted that the information provided from microscopy and metabarcoding complement each other^58,99^.

There are multiple other factors to consider in the selection of pollen analysis method(s) for pollinator-associated pollen including finances and available resources. Microscopy is the most accessible method in terms of finances and equipment, though the costs of high-throughput methods have been decreasing and many companies now offer metabarcoding services and core facilities with imaging flow cytometers exist in many larger cities (e.g. an overview for flow cytometry core facilities in Germany http://www.dgfz.org/dgfz2014/default_035.html). In sum, MIFC and metabarcoding can boost the analysis of large spatial and temporal scale pollination studies with their fast and automated processing.

## Conclusion

With the ongoing crises of climate change and biodiversity loss, there is increasing demand for monitoring their effects on the ecosystem service of pollination, alongside animal nutrition, across large spatial and temporal grains. Technical advancements in pollen identification and quantification are necessary to meet this demand in a resource-effective way. Our results evaluated the performance of several common and state-of-the art methods for pollen quantification, and found that high throughput methods have high potential for efficiently identifying pollen samples, with metabarcoding enabling highly accurate pollen identification and MIFC reliably assessing relative abundance, albeit contingent on currently available reference data.

## Supplementary Information

Below is the link to the electronic supplementary material.

Supplementary Material 1

Supplementary Material 2

## Data Availability

The datasets used in this study are accessible at 10.5281/zenodo.16787204.
