# Supplementary material for: Method comparison of microscopy, metabarcoding, and multispectral imaging flow cytometry for identification and relative abundance analysis of insect-dispersed pollen
Source: Sci Rep. 2026 Apr 16;16:12578. doi: 10.1038/s41598-026-47800-3 (PMC13087285; doi:10.1038/s41598-026-47800-3)
Supplement: Supplementary file 1 — Supplementary Material 1 [file 41598_2026_47800_MOESM1_ESM.docx]

**Supplemental figures**

Supplementary table S1. An overview of the mean number and standard error of pollen grains counted in a Neubauer improved counting chamber, with the number of chambers counted.

| Species | Mean (pollen grains) | Standard Error (SE) | Replicates (chambers) |
| --- | --- | --- | --- |
| *Centaurea phrygia* | 552 | 16 | 3 |
| *Hypericum perforatum* | 6866 | 792 | 4 |
| *Lathyrus pratensis* | 159 | 5 | 4 |
| *Leontodon hispidus* | 23 | 4 | 4 |
| *Lotus corniculatus* | 3213 | 255 | 4 |
| *Plantago lanceolata* | 286 | 36 | 4 |
| *Potentilla erecta* | 896 | 44 | 4 |
| *Prunella grandiflora* | 140 | 5 | 4 |
| *Stachys officinalis* | 332 | 48 | 4 |

Supplementary table S2. The set-up of the artificial pollen mixtures experiment with the proportion of species in the mixture adjusted according to the composition of the positive controls.

| **Species** | **Proportion of species in mixture (%)** | **Number of aliquots per method** | **Number of pollen grains per aliquot** |
| --- | --- | --- | --- |
| Treatment SmallDom: small pollen species dominating | | | |
| *Leontodon hispidus* | 0.421 | 10 | 5000 |
| *Lathyrus pratensis* | 1.122 |  |  |
| *Prunella grandiflora* | 1.142 |  |  |
| *Stachys officinalis* | 2.525 |  |  |
| *Centaurea phrygia* | 5.029 |  |  |
| *Plantago lanceolata* | 11.120 |  |  |
| *Potentilla erecta* | 15.187 |  |  |
| *Lotus corniculatus* | 26.027 |  |  |
| *Hypericum perforatum* | 37.427 |  |  |
| Treatment SmallInt: small pollen species intermediate | | | |
| *Leontodon hispidus* | 0.421 | 10 | 5000 |
| *Lathyrus pratensis* | 1.122 |  |  |
| *Prunella grandiflora* | 1.142 |  |  |
| *Potentilla erecta* | 2.504 |  |  |
| *Lotus corniculatus* | 5.089 |  |  |
| *Hypericum perforatum* | 11.962 |  |  |
| *Stachys officinalis* | 15.207 |  |  |
| *Centaurea phrygia* | 25.967 |  |  |
| *Plantago lanceolata* | 36.586 |  |  |
| Treatment SmallRar: small pollen species rare | | |  |
| *Lotus corniculatus* | 0.868 | 10 | 5000 |
| *Hypericum perforatum* | 1.272 |  |  |
| *Potentilla erecta* | 1.514 |  |  |
| *Leontodon hispidus* | 3.189 |  |  |
| *Lathyrus pratensis* | 4.521 |  |  |
| *Prunella grandiflora* | 10.133 |  |  |
| *Stachys officinalis* | 15.482 |  |  |
| *Centaurea phrygia* | 26.161 |  |  |
| *Plantago lanceolata* | 36.860 |  |  |

Supplementary table S3. The observed sample coverage calculated by method.

| Sample | Treatment | Type | Method | Observed sample coverage |
| --- | --- | --- | --- | --- |
| T1A1 | SmallDom | Artificial mixture | Microscopy | 0.9608 |
| T1A2 | SmallDom | Artificial mixture | Microscopy | 0.9362 |
| T1A3 | SmallDom | Artificial mixture | Microscopy | 0.9269 |
| T1A4 | SmallDom | Artificial mixture | Microscopy | 0.9636 |
| T1A5 | SmallDom | Artificial mixture | Microscopy | 1.0 |
| T1A6 | SmallDom | Artificial mixture | Microscopy | 0.9543 |
| T1A7 | SmallDom | Artificial mixture | Microscopy | 1.0 |
| T1A8 | SmallDom | Artificial mixture | Microscopy | 0.9269 |
| T1A9 | SmallDom | Artificial mixture | Microscopy | 0.9318 |
| T1A10 | SmallDom | Artificial mixture | Microscopy | 0.9531 |
| T2A1 | SmallInt | Artificial mixture | Microscopy | 0.9768 |
| T2A2 | SmallInt | Artificial mixture | Microscopy | 0.9841 |
| T2A3 | SmallInt | Artificial mixture | Microscopy | 1.0 |
| T2A4 | SmallInt | Artificial mixture | Microscopy | 0.963 |
| T2A5 | SmallInt | Artificial mixture | Microscopy | 1.0 |
| T2A6 | SmallInt | Artificial mixture | Microscopy | 0.9643 |
| T2A7 | SmallInt | Artificial mixture | Microscopy | 0.9498 |
| T2A8 | SmallInt | Artificial mixture | Microscopy | 0.9303 |
| T2A9 | SmallInt | Artificial mixture | Microscopy | 0.9461 |
| T2A10 | SmallInt | Artificial mixture | Microscopy | 0.96 |
| T3A1 | SmallRar | Artificial mixture | Microscopy | 0.9048 |
| T3A2 | SmallRar | Artificial mixture | Microscopy | 0.9815 |
| T3A3 | SmallRar | Artificial mixture | Microscopy | 1.0 |
| T3A4 | SmallRar | Artificial mixture | Microscopy | 0.889 |
| T3A5 | SmallRar | Artificial mixture | Microscopy | 1.0 |
| T3A6 | SmallRar | Artificial mixture | Microscopy | 0.9872 |
| T3A7 | SmallRar | Artificial mixture | Microscopy | 0.8734 |
| T3A8 | SmallRar | Artificial mixture | Microscopy | 0.9149 |
| T3A9 | SmallRar | Artificial mixture | Microscopy | 0.92 |
| T3A10 | SmallRar | Artificial mixture | Microscopy | 0.9503 |
| T1A1 | SmallDom | Artificial mixture | Metabarcoding | 1.0 |
| T1A2 | SmallDom | Artificial mixture | Metabarcoding | 1.0 |
| T1A3 | SmallDom | Artificial mixture | Metabarcoding | 1.0 |
| T1A4 | SmallDom | Artificial mixture | Metabarcoding | 1.0 |
| T1A5 | SmallDom | Artificial mixture | Metabarcoding | 1.0 |
| T1A6 | SmallDom | Artificial mixture | Metabarcoding | 1.0 |
| T1A7 | SmallDom | Artificial mixture | Metabarcoding | 1.0 |
| T1A8 | SmallDom | Artificial mixture | Metabarcoding | 1.0 |
| T1A9 | SmallDom | Artificial mixture | Metabarcoding | 1.0 |
| T1A10 | SmallDom | Artificial mixture | Metabarcoding | 1.0 |
| T2A1 | SmallInt | Artificial mixture | Metabarcoding | 1.0 |
| T2A2 | SmallInt | Artificial mixture | Metabarcoding | 1.0 |
| T2A3 | SmallInt | Artificial mixture | Metabarcoding | 1.0 |
| T2A4 | SmallInt | Artificial mixture | Metabarcoding | 1.0 |
| T2A5 | SmallInt | Artificial mixture | Metabarcoding | 1.0 |
| T2A6 | SmallInt | Artificial mixture | Metabarcoding | 1.0 |
| T2A7 | SmallInt | Artificial mixture | Metabarcoding | 1.0 |
| T2A8 | SmallInt | Artificial mixture | Metabarcoding | 1.0 |
| T2A9 | SmallInt | Artificial mixture | Metabarcoding | 1.0 |
| T2A10 | SmallInt | Artificial mixture | Metabarcoding | 1.0 |
| T3A1 | SmallRar | Artificial mixture | Metabarcoding | 1.0 |
| T3A2 | SmallRar | Artificial mixture | Metabarcoding | 1.0 |
| T3A3 | SmallRar | Artificial mixture | Metabarcoding | 1.0 |
| T3A4 | SmallRar | Artificial mixture | Metabarcoding | 1.0 |
| T3A5 | SmallRar | Artificial mixture | Metabarcoding | 1.0 |
| T3A6 | SmallRar | Artificial mixture | Metabarcoding | 1.0 |
| T3A7 | SmallRar | Artificial mixture | Metabarcoding | 1.0 |
| T3A8 | SmallRar | Artificial mixture | Metabarcoding | 1.0 |
| T3A9 | SmallRar | Artificial mixture | Metabarcoding | 1.0 |
| T3A10 | SmallRar | Artificial mixture | Metabarcoding | 1.0 |
| T1A1 | SmallDom | Artificial mixture | MIFC | 1.0 |
| T1A2 | SmallDom | Artificial mixture | MIFC | 1.0 |
| T1A3 | SmallDom | Artificial mixture | MIFC | 1.0 |
| T1A4 | SmallDom | Artificial mixture | MIFC | 1.0 |
| T1A5 | SmallDom | Artificial mixture | MIFC | 1.0 |
| T1A6 | SmallDom | Artificial mixture | MIFC | 1.0 |
| T1A7 | SmallDom | Artificial mixture | MIFC | 1.0 |
| T1A8 | SmallDom | Artificial mixture | MIFC | 1.0 |
| T1A9 | SmallDom | Artificial mixture | MIFC | 1.0 |
| T1A10 | SmallDom | Artificial mixture | MIFC | 1.0 |
| T2A1 | SmallInt | Artificial mixture | MIFC | 1.0 |
| T2A2 | SmallInt | Artificial mixture | MIFC | 1.0 |
| T2A3 | SmallInt | Artificial mixture | MIFC | 1.0 |
| T2A4 | SmallInt | Artificial mixture | MIFC | 1.0 |
| T2A5 | SmallInt | Artificial mixture | MIFC | 1.0 |
| T2A6 | SmallInt | Artificial mixture | MIFC | 1.0 |
| T2A7 | SmallInt | Artificial mixture | MIFC | 1.0 |
| T2A8 | SmallInt | Artificial mixture | MIFC | 1.0 |
| T2A9 | SmallInt | Artificial mixture | MIFC | 1.0 |
| T2A10 | SmallInt | Artificial mixture | MIFC | 1.0 |
| T3A1 | SmallRar | Artificial mixture | MIFC | 1.0 |
| T3A2 | SmallRar | Artificial mixture | MIFC | 1.0 |
| T3A3 | SmallRar | Artificial mixture | MIFC | 1.0 |
| T3A4 | SmallRar | Artificial mixture | MIFC | 1.0 |
| T3A5 | SmallRar | Artificial mixture | MIFC | 1.0 |
| T3A6 | SmallRar | Artificial mixture | MIFC | 1.0 |
| T3A7 | SmallRar | Artificial mixture | MIFC | 1.0 |
| T3A8 | SmallRar | Artificial mixture | MIFC | 1.0 |
| T3A9 | SmallRar | Artificial mixture | MIFC | 1.0 |
| T3A10 | SmallRar | Artificial mixture | MIFC | 1.0 |
| Bumble bee 1 | - | Insect pollen | Microscopy | 1.0 |
| Bumble bee 2 | - | Insect pollen | Microscopy | 1.0 |
| Bumble bee 3 | - | Insect pollen | Microscopy | 0.9396 |
| Bumble bee 4 | - | Insect pollen | Microscopy | 0.9794 |
| Bumble bee 5 | - | Insect pollen | Microscopy | 1.0 |
| Bumble bee 6 | - | Insect pollen | Microscopy | 1.0 |
| Bumble bee 7 | - | Insect pollen | Microscopy | 1.0 |
| Bumble bee 8 | - | Insect pollen | Microscopy | 1.0 |
| Bumble bee 9 | - | Insect pollen | Microscopy | 1.0 |
| Bumble bee 10 | - | Insect pollen | Microscopy | 1.0 |
| Fly 1 | - | Insect pollen | Microscopy | 1.0 |
| Fly 2 | - | Insect pollen | Microscopy | 1.0 |
| Fly 3 | - | Insect pollen | Microscopy | 1.0 |
| Fly 4 | - | Insect pollen | Microscopy | 1.0 |
| Fly 5 | - | Insect pollen | Microscopy | 1.0 |
| Fly 6 | - | Insect pollen | Microscopy | 1.0 |
| Fly 7 | - | Insect pollen | Microscopy | 1.0 |
| Fly 8 | - | Insect pollen | Microscopy | 1.0 |
| Fly 9 | - | Insect pollen | Microscopy | 1.0 |
| Fly 10 | - | Insect pollen | Microscopy | 1.0 |
| Wild bee 1 | - | Insect pollen | Microscopy | 1.0 |
| Wild bee 2 | - | Insect pollen | Microscopy | 1.0 |
| Wild bee 3 | - | Insect pollen | Microscopy | 0.9723 |
| Wild bee 4 | - | Insect pollen | Microscopy | 0.9546 |
| Wild bee 5 | - | Insect pollen | Microscopy | 0.9305 |
| Wild bee 6 | - | Insect pollen | Microscopy | 1.0 |
| Wild bee 7 | - | Insect pollen | Microscopy | 1.0 |
| Wild bee 8 | - | Insect pollen | Microscopy | 0.9688 |
| Wild bee 9 | - | Insect pollen | Microscopy | 1.0 |
| Wild bee 10 | - | Insect pollen | Microscopy | 1.0 |
| Bumble bee 1 | - | Insect pollen | Metabarcoding | 1.0 |
| Bumble bee 2 | - | Insect pollen | Metabarcoding | 1.0 |
| Bumble bee 3 | - | Insect pollen | Metabarcoding | 1.0 |
| Bumble bee 4 | - | Insect pollen | Metabarcoding | 1.0 |
| Bumble bee 5 | - | Insect pollen | Metabarcoding | 1.0 |
| Bumble bee 6 | - | Insect pollen | Metabarcoding | 1.0 |
| Bumble bee 7 | - | Insect pollen | Metabarcoding | 1.0 |
| Bumble bee 8 | - | Insect pollen | Metabarcoding | 1.0 |
| Bumble bee 9 | - | Insect pollen | Metabarcoding | 1.0 |
| Bumble bee 10 | - | Insect pollen | Metabarcoding | 1.0 |
| Fly 1 | - | Insect pollen | Metabarcoding | 1.0 |
| Fly 2 | - | Insect pollen | Metabarcoding | 1.0 |
| Fly 3 | - | Insect pollen | Metabarcoding | 1.0 |
| Fly 4 | - | Insect pollen | Metabarcoding | 1.0 |
| Fly 5 | - | Insect pollen | Metabarcoding | 1.0 |
| Fly 6 | - | Insect pollen | Metabarcoding | 1.0 |
| Fly 7 | - | Insect pollen | Metabarcoding | 1.0 |
| Fly 8 | - | Insect pollen | Metabarcoding | 1.0 |
| Fly 9 | - | Insect pollen | Metabarcoding | 1.0 |
| Fly 10 | - | Insect pollen | Metabarcoding | 1.0 |
| Wild bee 1 | - | Insect pollen | Metabarcoding | 1.0 |
| Wild bee 2 | - | Insect pollen | Metabarcoding | 1.0 |
| Wild bee 3 | - | Insect pollen | Metabarcoding | 1.0 |
| Wild bee 4 | - | Insect pollen | Metabarcoding | 1.0 |
| Wild bee 5 | - | Insect pollen | Metabarcoding | 1.0 |
| Wild bee 6 | - | Insect pollen | Metabarcoding | 1.0 |
| Wild bee 7 | - | Insect pollen | Metabarcoding | 1.0 |
| Wild bee 8 | - | Insect pollen | Metabarcoding | 1.0 |
| Wild bee 9 | - | Insect pollen | Metabarcoding | 1.0 |
| Wild bee 10 | - | Insect pollen | Metabarcoding | 1.0 |
| Bumble bee 1 | - | Insect pollen | MIFC | 1.0 |
| Bumble bee 2 | - | Insect pollen | MIFC | 1.0 |
| Bumble bee 3 | - | Insect pollen | MIFC | 1.0 |
| Bumble bee 4 | - | Insect pollen | MIFC | 1.0 |
| Bumble bee 5 | - | Insect pollen | MIFC | 1.0 |
| Bumble bee 6 | - | Insect pollen | MIFC | 1.0 |
| Bumble bee 7 | - | Insect pollen | MIFC | 1.0 |
| Bumble bee 8 | - | Insect pollen | MIFC | 1.0 |
| Bumble bee 9 | - | Insect pollen | MIFC | 1.0 |
| Bumble bee 10 | - | Insect pollen | MIFC | 1.0 |
| Fly 1 | - | Insect pollen | MIFC | 1.0 |
| Fly 2 | - | Insect pollen | MIFC | 1.0 |
| Fly 3 | - | Insect pollen | MIFC | 1.0 |
| Fly 4 | - | Insect pollen | MIFC | 1.0 |
| Fly 5 | - | Insect pollen | MIFC | 1.0 |
| Fly 6 | - | Insect pollen | MIFC | 1.0 |
| Fly 7 | - | Insect pollen | MIFC | 1.0 |
| Fly 8 | - | Insect pollen | MIFC | 1.0 |
| Fly 9 | - | Insect pollen | MIFC | 1.0 |
| Fly 10 | - | Insect pollen | MIFC | 1.0 |
| Wild bee 1 | - | Insect pollen | MIFC | 1.0 |
| Wild bee 2 | - | Insect pollen | MIFC | 1.0 |
| Wild bee 3 | - | Insect pollen | MIFC | 1.0 |
| Wild bee 4 | - | Insect pollen | MIFC | 1.0 |
| Wild bee 5 | - | Insect pollen | MIFC | 1.0 |
| Wild bee 6 | - | Insect pollen | MIFC | 1.0 |
| Wild bee 7 | - | Insect pollen | MIFC | 1.0 |
| Wild bee 8 | - | Insect pollen | MIFC | 1.0 |
| Wild bee 9 | - | Insect pollen | MIFC | 1.0 |
| Wild bee 10 | - | Insect pollen | MIFC | 1.0 |

Supplementary figure S4. The sample coverage shown per number of individuals (pollen grains/reads) for the artificial mixtures (T1: SmallDom, T2: SmallInt, T3; SmallRar) a) MIFC, b) metabarcoding, c) microscopy and insect samples d) MIFC, e) metabarcoding, and f) microscopy.


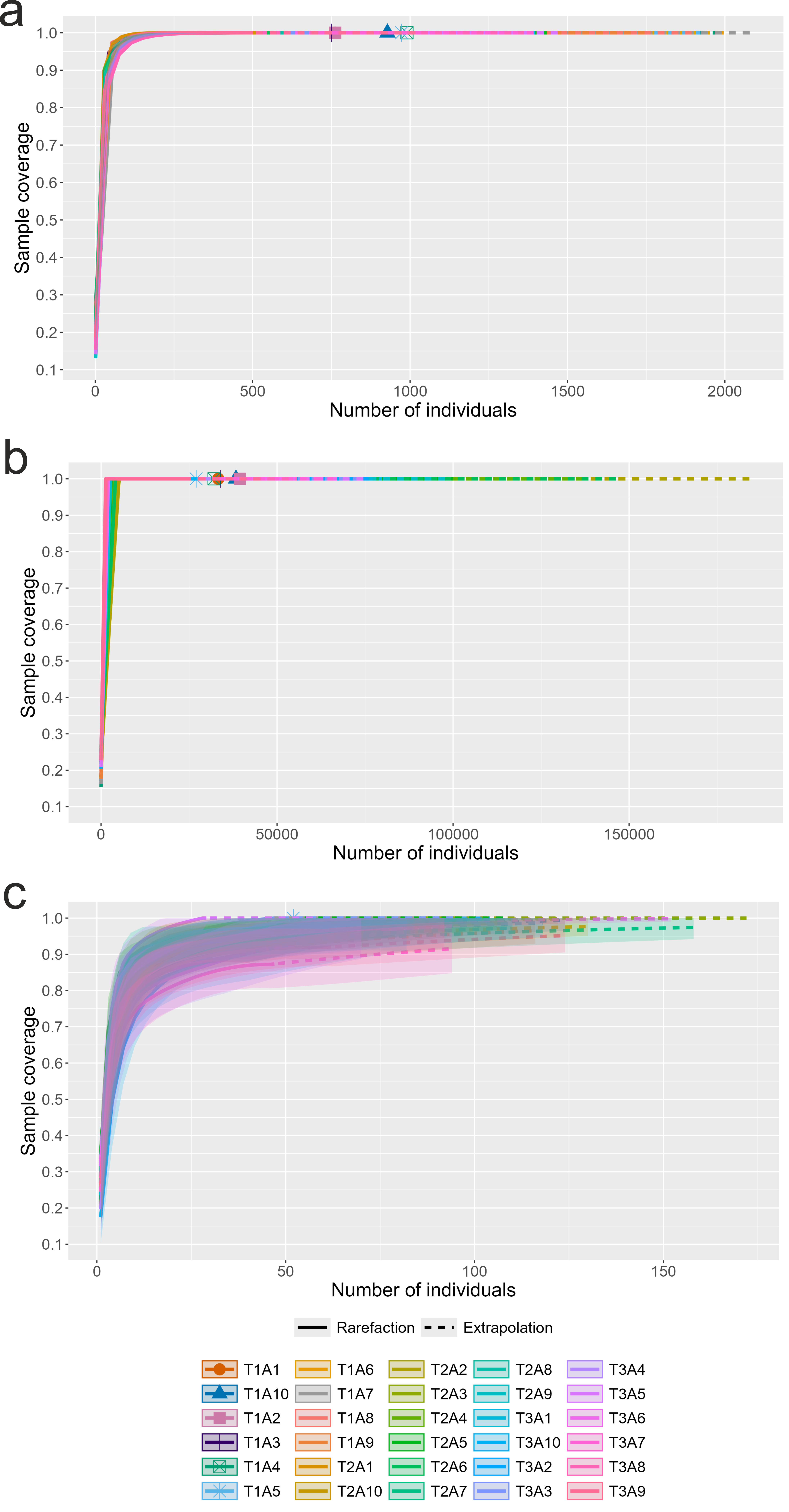

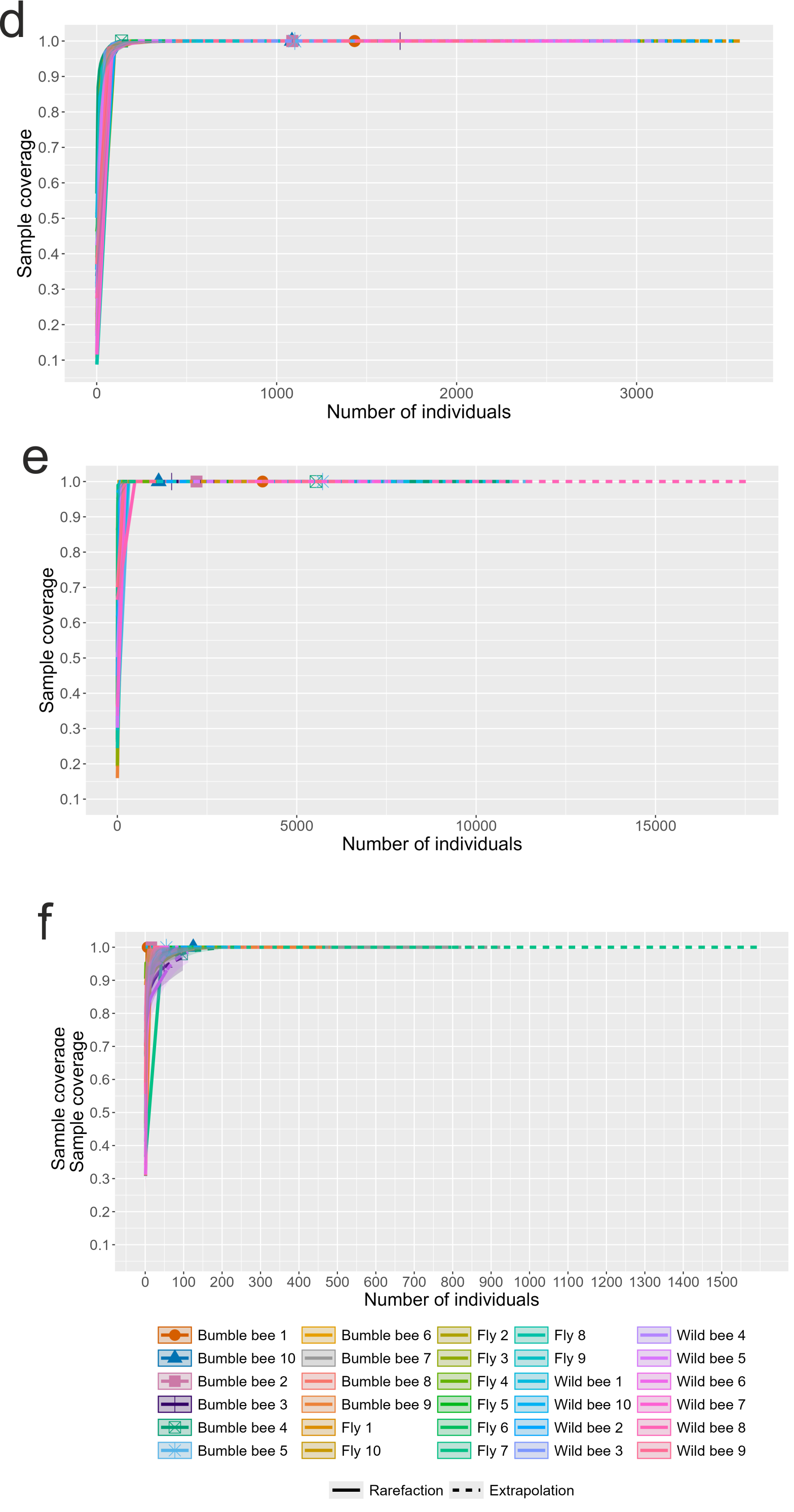


Supplementary table S5. Summary statistics of Kruskal-Wallis rank sum tests and subsequent pairwise Wilcoxon “Holm”-adjusted post-hoc tests on the proportion of taxa (that were put into the mixture) detected for each treatment compared at the genus level.

| Test | Treatment | Comparison | Kruskal-Wallis chi-squared | df | p-value |
| --- | --- | --- | --- | --- | --- |
| Kruskal-Wallis rank sum test | SmallDom | Proportion of genera detected by method | 26.979 | 2 | 1.385e-06 |
| Wilcoxon rank sum test with continuity correction | SmallDom | MIFC- Metabarcoding | - | - | 0.00014 |
| Wilcoxon rank sum test with continuity correction | SmallDom | MIFC-Microscopy | - | - | 0.00014 |
| Wilcoxon rank sum test with continuity correction | SmallDom | Metabarcoding-Microscopy | - | - | 0.00014 |
| Kruskal-Wallis rank sum test | SmallInt | Proportion of genera detected by method | 19.372 | 2 | 6.214e-05 |
| Wilcoxon rank sum test with continuity correction | SmallInt | MIFC- Metabarcoding | - | - | 0.00061 |
| Wilcoxon rank sum test with continuity correction | SmallInt | MIFC-Microscopy | - | - | 0.41288 |
| Wilcoxon rank sum test with continuity correction | SmallInt | Metabarcoding-Microscopy | - | - | 0.00061 |
| Kruskal-Wallis rank sum test | SmallRar | Proportion of genera detected by method | 23.673 | 2 | 7.236e-06 |
| Wilcoxon rank sum test with continuity correction | SmallRar | MIFC- Metabarcoding | - | - | 0.00021 |
| Wilcoxon rank sum test with continuity correction | SmallRar | MIFC-Microscopy | - | - | 0.00809 |
| Wilcoxon rank sum test with continuity correction | SmallRar | Metabarcoding-Microscopy | - | - | 0.00021 |

Supplementary table S6. The counts of the nine original genera that were found by each method is in the following file: Supplementary_Table_S6.csv

Supplementary figure S7. Linear regression lines show the variability of the identification results of each method explained by the true proportions (1:1 line) for the “blind” analysis for a) Treatment SmallDom, b) Treatment SmallInt, c) Treatment SmallRar, and d) all treatments combined.


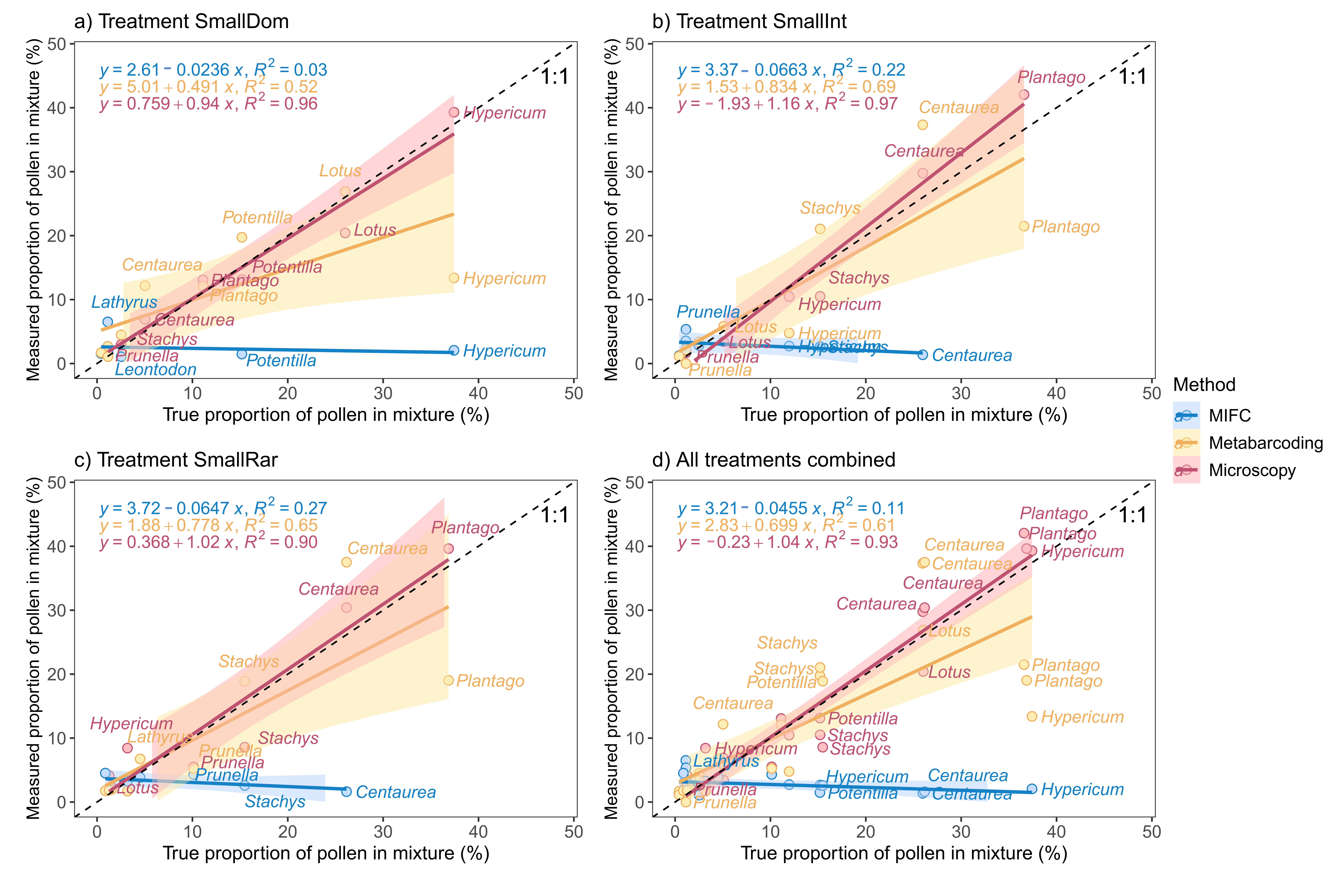


Supplemental table S8. Summary statistics of Kruskal-Wallis rank sum test to describe the significance differences of the relative proportions of each species in each mixture between the three methods for the “informed” analysis.

| Treatment | Species | n | Kruskal-wallis chi-squared | df | p-value |
| --- | --- | --- | --- | --- | --- |
| Treatment SmallDom | *Centaurea phrygia* | 40 | 29.38805 | 3 | 1.86E-06 |
| Treatment SmallDom | *Hypericum perforatum* | 42 | 26.97629 | 3 | 5.96E-06 |
| Treatment SmallDom | *Lotus corniculatus* | 40 | 27.80204 | 3 | 4.00E-06 |
| Treatment SmallDom | *Leontodon hispidus* | 37 | 30.54774 | 3 | 1.06E-06 |
| Treatment SmallDom | *Lathyrus pratensis* | 34 | 27.09466 | 3 | 5.62E-06 |
| Treatment SmallDom | *Potentilla erecta* | 40 | 32.71778 | 3 | 3.69E-07 |
| Treatment SmallDom | *Prunella grandiflora* | 24 | 14.11457 | 3 | 0.002753 |
| Treatment SmallDom | *Plantago lanceolata* | 40 | 12.62121 | 3 | 0.005532 |
| Treatment SmallDom | *Stachys officinalis* | 42 | 22.11406 | 3 | 6.18E-05 |
| Treatment SmallInt | *Centaurea phrygia* | 40 | 33.92758 | 3 | 2.05E-07 |
| Treatment SmallInt | *Hypericum perforatum* | 40 | 30.91609 | 3 | 8.85E-07 |
| Treatment SmallInt | *Lotus corniculatus* | 38 | 16.64299 | 3 | 0.000837 |
| Treatment SmallInt | *Leontodon hispidus* | 29 | 24.34001 | 3 | 2.12E-05 |
| Treatment SmallInt | *Lathyrus pratensis* | 31 | 19.98332 | 3 | 0.000171 |
| Treatment SmallInt | *Potentilla erecta* | 38 | 14.68641 | 3 | 0.002105 |
| Treatment SmallInt | *Prunella grandiflora* | 26 | 22.80254 | 3 | 4.44E-05 |
| Treatment SmallInt | *Plantago lanceolata* | 40 | 33.04465 | 3 | 3.15E-07 |
| Treatment SmallInt | *Stachys officinalis* | 40 | 32.22722 | 3 | 4.69E-07 |
| Treatment SmallRar | *Centaurea phrygia* | 40 | 32.05321 | 3 | 5.10E-07 |
| Treatment SmallRar | *Hypericum perforatum* | 39 | 26.39543 | 3 | 7.88E-06 |
| Treatment SmallRar | *Lotus corniculatus* | 33 | 23.23093 | 3 | 3.61E-05 |
| Treatment SmallRar | *Leontodon hispidus* | 39 | 28.35522 | 3 | 3.06E-06 |
| Treatment SmallRar | *Lathyrus pratensis* | 37 | 25.63518 | 3 | 1.14E-05 |
| Treatment SmallRar | *Potentilla erecta* | 36 | 24.84716 | 3 | 1.66E-05 |
| Treatment SmallRar | *Prunella grandiflora* | 41 | 34.95968 | 3 | 1.24E-07 |
| Treatment SmallRar | *Plantago lanceolata* | 40 | 32.75926 | 3 | 3.62E-07 |
| Treatment SmallRar | *Stachys officinalis* | 40 | 32.08739 | 3 | 5.02E-07 |

Supplemental table S9. Summary statistics of pairwise Wilcoxon “Holm”-adjusted post-hoc tests to describe the significance levels of the relative proportions of each species in each mixture between the three methods for the “informed” analysis.

| Treatment | Species | Method 1 | Method 2 | p-value |
| --- | --- | --- | --- | --- |
| Treatment SmallDom | *Centaurea phrygia* | Metabarcoding | Flow cytometry | 6.50E-05 |
| Treatment SmallDom | *Centaurea phrygia* | Microscopy | Flow cytometry | 0.057611 |
| Treatment SmallDom | *Centaurea phrygia* | True proportion | Flow cytometry | 0.000255 |
| Treatment SmallDom | *Centaurea phrygia* | Metabarcoding | Metabarcoding | NA |
| Treatment SmallDom | *Centaurea phrygia* | Microscopy | Metabarcoding | 6.50E-05 |
| Treatment SmallDom | *Centaurea phrygia* | True proportion | Metabarcoding | 0.000255 |
| Treatment SmallDom | *Centaurea phrygia* | Metabarcoding | Microscopy | NA |
| Treatment SmallDom | *Centaurea phrygia* | Microscopy | Microscopy | NA |
| Treatment SmallDom | *Centaurea phrygia* | True proportion | Microscopy | 0.115258 |
| Treatment SmallDom | *Hypericum perforatum* | Metabarcoding | Flow cytometry | 6.50E-05 |
| Treatment SmallDom | *Hypericum perforatum* | Microscopy | Flow cytometry | 0.010309 |
| Treatment SmallDom | *Hypericum perforatum* | True proportion | Flow cytometry | 0.000319 |
| Treatment SmallDom | *Hypericum perforatum* | Metabarcoding | Metabarcoding | NA |
| Treatment SmallDom | *Hypericum perforatum* | Microscopy | Metabarcoding | 0.014289 |
| Treatment SmallDom | *Hypericum perforatum* | True proportion | Metabarcoding | 0.000319 |
| Treatment SmallDom | *Hypericum perforatum* | Metabarcoding | Microscopy | NA |
| Treatment SmallDom | *Hypericum perforatum* | Microscopy | Microscopy | NA |
| Treatment SmallDom | *Hypericum perforatum* | True proportion | Microscopy | 1 |
| Treatment SmallDom | *Lotus corniculatus* | Metabarcoding | Flow cytometry | 6.50E-05 |
| Treatment SmallDom | *Lotus corniculatus* | Microscopy | Flow cytometry | 0.393048 |
| Treatment SmallDom | *Lotus corniculatus* | True proportion | Flow cytometry | 0.000319 |
| Treatment SmallDom | *Lotus corniculatus* | Metabarcoding | Metabarcoding | NA |
| Treatment SmallDom | *Lotus corniculatus* | Microscopy | Metabarcoding | 0.004263 |
| Treatment SmallDom | *Lotus corniculatus* | True proportion | Metabarcoding | 0.000319 |
| Treatment SmallDom | *Lotus corniculatus* | Metabarcoding | Microscopy | NA |
| Treatment SmallDom | *Lotus corniculatus* | Microscopy | Microscopy | NA |
| Treatment SmallDom | *Lotus corniculatus* | True proportion | Microscopy | 0.004263 |
| Treatment SmallDom | *Leontodon hispidus* | Metabarcoding | Flow cytometry | 0.001451 |
| Treatment SmallDom | *Leontodon hispidus* | Microscopy | Flow cytometry | 0.000654 |
| Treatment SmallDom | *Leontodon hispidus* | True proportion | Flow cytometry | 0.000383 |
| Treatment SmallDom | *Leontodon hispidus* | Metabarcoding | Metabarcoding | NA |
| Treatment SmallDom | *Leontodon hispidus* | Microscopy | Metabarcoding | 0.108803 |
| Treatment SmallDom | *Leontodon hispidus* | True proportion | Metabarcoding | 0.000383 |
| Treatment SmallDom | *Leontodon hispidus* | Metabarcoding | Microscopy | NA |
| Treatment SmallDom | *Leontodon hispidus* | Microscopy | Microscopy | NA |
| Treatment SmallDom | *Leontodon hispidus* | True proportion | Microscopy | 0.000654 |
| Treatment SmallDom | *Lathyrus pratensis* | Metabarcoding | Flow cytometry | 6.50E-05 |
| Treatment SmallDom | *Lathyrus pratensis* | Microscopy | Flow cytometry | 0.005994 |
| Treatment SmallDom | *Lathyrus pratensis* | True proportion | Flow cytometry | 0.034374 |
| Treatment SmallDom | *Lathyrus pratensis* | Metabarcoding | Metabarcoding | NA |
| Treatment SmallDom | *Lathyrus pratensis* | Microscopy | Metabarcoding | 0.075924 |
| Treatment SmallDom | *Lathyrus pratensis* | True proportion | Metabarcoding | 0.000319 |
| Treatment SmallDom | *Lathyrus pratensis* | Metabarcoding | Microscopy | NA |
| Treatment SmallDom | *Lathyrus pratensis* | Microscopy | Microscopy | NA |
| Treatment SmallDom | *Lathyrus pratensis* | True proportion | Microscopy | 0.002207 |
| Treatment SmallDom | *Potentilla erecta* | Metabarcoding | Flow cytometry | 6.50E-05 |
| Treatment SmallDom | *Potentilla erecta* | Microscopy | Flow cytometry | 0.011554 |
| Treatment SmallDom | *Potentilla erecta* | True proportion | Flow cytometry | 0.000319 |
| Treatment SmallDom | *Potentilla erecta* | Metabarcoding | Metabarcoding | NA |
| Treatment SmallDom | *Potentilla erecta* | Microscopy | Metabarcoding | 0.000545 |
| Treatment SmallDom | *Potentilla erecta* | True proportion | Metabarcoding | 0.000319 |
| Treatment SmallDom | *Potentilla erecta* | Metabarcoding | Microscopy | NA |
| Treatment SmallDom | *Potentilla erecta* | Microscopy | Microscopy | NA |
| Treatment SmallDom | *Potentilla erecta* | True proportion | Microscopy | 0.017139 |
| Treatment SmallDom | *Prunella grandiflora* | Metabarcoding | Flow cytometry | 1 |
| Treatment SmallDom | *Prunella grandiflora* | Microscopy | Flow cytometry | 1 |
| Treatment SmallDom | *Prunella grandiflora* | True proportion | Flow cytometry | 0.007105 |
| Treatment SmallDom | *Prunella grandiflora* | Metabarcoding | Metabarcoding | NA |
| Treatment SmallDom | *Prunella grandiflora* | Microscopy | Metabarcoding | 1 |
| Treatment SmallDom | *Prunella grandiflora* | True proportion | Metabarcoding | 0.017706 |
| Treatment SmallDom | *Prunella grandiflora* | Metabarcoding | Microscopy | NA |
| Treatment SmallDom | *Prunella grandiflora* | Microscopy | Microscopy | NA |
| Treatment SmallDom | *Prunella grandiflora* | True proportion | Microscopy | 0.005502 |
| Treatment SmallDom | *Plantago lanceolata* | Metabarcoding | Flow cytometry | 1 |
| Treatment SmallDom | *Plantago lanceolata* | Microscopy | Flow cytometry | 1 |
| Treatment SmallDom | *Plantago lanceolata* | True proportion | Flow cytometry | 0.000383 |
| Treatment SmallDom | *Plantago lanceolata* | Metabarcoding | Metabarcoding | NA |
| Treatment SmallDom | *Plantago lanceolata* | Microscopy | Metabarcoding | 1 |
| Treatment SmallDom | *Plantago lanceolata* | True proportion | Metabarcoding | 0.000383 |
| Treatment SmallDom | *Plantago lanceolata* | Metabarcoding | Microscopy | NA |
| Treatment SmallDom | *Plantago lanceolata* | Microscopy | Microscopy | NA |
| Treatment SmallDom | *Plantago lanceolata* | True proportion | Microscopy | 1 |
| Treatment SmallDom | *Stachys officinalis* | Metabarcoding | Flow cytometry | 6.50E-05 |
| Treatment SmallDom | *Stachys officinalis* | Microscopy | Flow cytometry | 0.868846 |
| Treatment SmallDom | *Stachys officinalis* | True proportion | Flow cytometry | 0.005684 |
| Treatment SmallDom | *Stachys officinalis* | Metabarcoding | Metabarcoding | NA |
| Treatment SmallDom | *Stachys officinalis* | Microscopy | Metabarcoding | 0.018502 |
| Treatment SmallDom | *Stachys officinalis* | True proportion | Metabarcoding | 0.000319 |
| Treatment SmallDom | *Stachys officinalis* | Metabarcoding | Microscopy | NA |
| Treatment SmallDom | *Stachys officinalis* | Microscopy | Microscopy | NA |
| Treatment SmallDom | *Stachys officinalis* | True proportion | Microscopy | 0.081796 |
| Treatment SmallInt | *Centaurea phrygia* | Metabarcoding | Flow cytometry | 6.50E-05 |
| Treatment SmallInt | *Centaurea phrygia* | Microscopy | Flow cytometry | 6.50E-05 |
| Treatment SmallInt | *Centaurea phrygia* | True proportion | Flow cytometry | 0.000255 |
| Treatment SmallInt | *Centaurea phrygia* | Metabarcoding | Metabarcoding | NA |
| Treatment SmallInt | *Centaurea phrygia* | Microscopy | Metabarcoding | 0.000411 |
| Treatment SmallInt | *Centaurea phrygia* | True proportion | Metabarcoding | 0.000255 |
| Treatment SmallInt | *Centaurea phrygia* | Metabarcoding | Microscopy | NA |
| Treatment SmallInt | *Centaurea phrygia* | Microscopy | Microscopy | NA |
| Treatment SmallInt | *Centaurea phrygia* | True proportion | Microscopy | 0.017187 |
| Treatment SmallInt | *Hypericum perforatum* | Metabarcoding | Flow cytometry | 6.50E-05 |
| Treatment SmallInt | *Hypericum perforatum* | Microscopy | Flow cytometry | 0.000319 |
| Treatment SmallInt | *Hypericum perforatum* | True proportion | Flow cytometry | 0.000319 |
| Treatment SmallInt | *Hypericum perforatum* | Metabarcoding | Metabarcoding | NA |
| Treatment SmallInt | *Hypericum perforatum* | Microscopy | Metabarcoding | 0.003009 |
| Treatment SmallInt | *Hypericum perforatum* | True proportion | Metabarcoding | 0.000319 |
| Treatment SmallInt | *Hypericum perforatum* | Metabarcoding | Microscopy | NA |
| Treatment SmallInt | *Hypericum perforatum* | Microscopy | Microscopy | NA |
| Treatment SmallInt | *Hypericum perforatum* | True proportion | Microscopy | 0.442901 |
| Treatment SmallInt | *Lotus corniculatus* | Metabarcoding | Flow cytometry | 0.630529 |
| Treatment SmallInt | *Lotus corniculatus* | Microscopy | Flow cytometry | 0.024864 |
| Treatment SmallInt | *Lotus corniculatus* | True proportion | Flow cytometry | 0.008525 |
| Treatment SmallInt | *Lotus corniculatus* | Metabarcoding | Metabarcoding | NA |
| Treatment SmallInt | *Lotus corniculatus* | Microscopy | Metabarcoding | 0.024864 |
| Treatment SmallInt | *Lotus corniculatus* | True proportion | Metabarcoding | 0.034374 |
| Treatment SmallInt | *Lotus corniculatus* | Metabarcoding | Microscopy | NA |
| Treatment SmallInt | *Lotus corniculatus* | Microscopy | Microscopy | NA |
| Treatment SmallInt | *Lotus corniculatus* | True proportion | Microscopy | 0.020038 |
| Treatment SmallInt | *Leontodon hispidus* | Metabarcoding | Flow cytometry | 0.254412 |
| Treatment SmallInt | *Leontodon hispidus* | Microscopy | Flow cytometry | 0.017301 |
| Treatment SmallInt | *Leontodon hispidus* | True proportion | Flow cytometry | 0.000383 |
| Treatment SmallInt | *Leontodon hispidus* | Metabarcoding | Metabarcoding | NA |
| Treatment SmallInt | *Leontodon hispidus* | Microscopy | Metabarcoding | 0.038902 |
| Treatment SmallInt | *Leontodon hispidus* | True proportion | Metabarcoding | 0.001766 |
| Treatment SmallInt | *Leontodon hispidus* | Metabarcoding | Microscopy | NA |
| Treatment SmallInt | *Leontodon hispidus* | Microscopy | Microscopy | NA |
| Treatment SmallInt | *Leontodon hispidus* | True proportion | Microscopy | 0.002159 |
| Treatment SmallInt | *Lathyrus pratensis* | Metabarcoding | Flow cytometry | 0.014397 |
| Treatment SmallInt | *Lathyrus pratensis* | Microscopy | Flow cytometry | 0.727273 |
| Treatment SmallInt | *Lathyrus pratensis* | True proportion | Flow cytometry | 0.051561 |
| Treatment SmallInt | *Lathyrus pratensis* | Metabarcoding | Metabarcoding | NA |
| Treatment SmallInt | *Lathyrus pratensis* | Microscopy | Metabarcoding | 0.727273 |
| Treatment SmallInt | *Lathyrus pratensis* | True proportion | Metabarcoding | 0.000383 |
| Treatment SmallInt | *Lathyrus pratensis* | Metabarcoding | Microscopy | NA |
| Treatment SmallInt | *Lathyrus pratensis* | Microscopy | Microscopy | NA |
| Treatment SmallInt | *Lathyrus pratensis* | True proportion | Microscopy | 0.017706 |
| Treatment SmallInt | *Potentilla erecta* | Metabarcoding | Flow cytometry | 0.002436 |
| Treatment SmallInt | *Potentilla erecta* | Microscopy | Flow cytometry | 1 |
| Treatment SmallInt | *Potentilla erecta* | True proportion | Flow cytometry | 0.068748 |
| Treatment SmallInt | *Potentilla erecta* | Metabarcoding | Metabarcoding | NA |
| Treatment SmallInt | *Potentilla erecta* | Microscopy | Metabarcoding | 0.94625 |
| Treatment SmallInt | *Potentilla erecta* | True proportion | Metabarcoding | 0.000383 |
| Treatment SmallInt | *Potentilla erecta* | Metabarcoding | Microscopy | NA |
| Treatment SmallInt | *Potentilla erecta* | Microscopy | Microscopy | NA |
| Treatment SmallInt | *Potentilla erecta* | True proportion | Microscopy | 1 |
| Treatment SmallInt | *Prunella grandiflora* | Metabarcoding | Flow cytometry | 0.363636 |
| Treatment SmallInt | *Prunella grandiflora* | Microscopy | Flow cytometry | 0.010656 |
| Treatment SmallInt | *Prunella grandiflora* | True proportion | Flow cytometry | 0.000383 |
| Treatment SmallInt | *Prunella grandiflora* | Metabarcoding | Metabarcoding | NA |
| Treatment SmallInt | *Prunella grandiflora* | Microscopy | Metabarcoding | 0.363636 |
| Treatment SmallInt | *Prunella grandiflora* | True proportion | Metabarcoding | 0.01328 |
| Treatment SmallInt | *Prunella grandiflora* | Metabarcoding | Microscopy | NA |
| Treatment SmallInt | *Prunella grandiflora* | Microscopy | Microscopy | NA |
| Treatment SmallInt | *Prunella grandiflora* | True proportion | Microscopy | 0.001766 |
| Treatment SmallInt | *Plantago lanceolata* | Metabarcoding | Flow cytometry | 6.50E-05 |
| Treatment SmallInt | *Plantago lanceolata* | Microscopy | Flow cytometry | 0.028806 |
| Treatment SmallInt | *Plantago lanceolata* | True proportion | Flow cytometry | 0.000255 |
| Treatment SmallInt | *Plantago lanceolata* | Metabarcoding | Metabarcoding | NA |
| Treatment SmallInt | *Plantago lanceolata* | Microscopy | Metabarcoding | 6.50E-05 |
| Treatment SmallInt | *Plantago lanceolata* | True proportion | Metabarcoding | 0.000255 |
| Treatment SmallInt | *Plantago lanceolata* | Metabarcoding | Microscopy | NA |
| Treatment SmallInt | *Plantago lanceolata* | Microscopy | Microscopy | NA |
| Treatment SmallInt | *Plantago lanceolata* | True proportion | Microscopy | 0.002842 |
| Treatment SmallInt | *Stachys officinalis* | Metabarcoding | Flow cytometry | 6.50E-05 |
| Treatment SmallInt | *Stachys officinalis* | Microscopy | Flow cytometry | 0.025692 |
| Treatment SmallInt | *Stachys officinalis* | True proportion | Flow cytometry | 0.000319 |
| Treatment SmallInt | *Stachys officinalis* | Metabarcoding | Metabarcoding | NA |
| Treatment SmallInt | *Stachys officinalis* | Microscopy | Metabarcoding | 0.001312 |
| Treatment SmallInt | *Stachys officinalis* | True proportion | Metabarcoding | 0.000319 |
| Treatment SmallInt | *Stachys officinalis* | Metabarcoding | Microscopy | NA |
| Treatment SmallInt | *Stachys officinalis* | Microscopy | Microscopy | NA |
| Treatment SmallInt | *Stachys officinalis* | True proportion | Microscopy | 0.002828 |
| Treatment SmallRar | *Centaurea phrygia* | Metabarcoding | Flow cytometry | 6.50E-05 |
| Treatment SmallRar | *Centaurea phrygia* | Microscopy | Flow cytometry | 6.50E-05 |
| Treatment SmallRar | *Centaurea phrygia* | True proportion | Flow cytometry | 0.000255 |
| Treatment SmallRar | *Centaurea phrygia* | Metabarcoding | Metabarcoding | NA |
| Treatment SmallRar | *Centaurea phrygia* | Microscopy | Metabarcoding | 0.029379 |
| Treatment SmallRar | *Centaurea phrygia* | True proportion | Metabarcoding | 0.000255 |
| Treatment SmallRar | *Centaurea phrygia* | Metabarcoding | Microscopy | NA |
| Treatment SmallRar | *Centaurea phrygia* | Microscopy | Microscopy | NA |
| Treatment SmallRar | *Centaurea phrygia* | True proportion | Microscopy | 0.029379 |
| Treatment SmallRar | *Hypericum perforatum* | Metabarcoding | Flow cytometry | 0.00013 |
| Treatment SmallRar | *Hypericum perforatum* | Microscopy | Flow cytometry | 0.795936 |
| Treatment SmallRar | *Hypericum perforatum* | True proportion | Flow cytometry | 0.000319 |
| Treatment SmallRar | *Hypericum perforatum* | Metabarcoding | Metabarcoding | NA |
| Treatment SmallRar | *Hypericum perforatum* | Microscopy | Metabarcoding | 0.001949 |
| Treatment SmallRar | *Hypericum perforatum* | True proportion | Metabarcoding | 0.000341 |
| Treatment SmallRar | *Hypericum perforatum* | Metabarcoding | Microscopy | NA |
| Treatment SmallRar | *Hypericum perforatum* | Microscopy | Microscopy | NA |
| Treatment SmallRar | *Hypericum perforatum* | True proportion | Microscopy | 0.034374 |
| Treatment SmallRar | *Lotus corniculatus* | Metabarcoding | Flow cytometry | 0.106389 |
| Treatment SmallRar | *Lotus corniculatus* | Microscopy | Flow cytometry | 0.741259 |
| Treatment SmallRar | *Lotus corniculatus* | True proportion | Flow cytometry | 0.000383 |
| Treatment SmallRar | *Lotus corniculatus* | Metabarcoding | Metabarcoding | NA |
| Treatment SmallRar | *Lotus corniculatus* | Microscopy | Metabarcoding | 0.741259 |
| Treatment SmallRar | *Lotus corniculatus* | True proportion | Metabarcoding | 0.000383 |
| Treatment SmallRar | *Lotus corniculatus* | Metabarcoding | Microscopy | NA |
| Treatment SmallRar | *Lotus corniculatus* | Microscopy | Microscopy | NA |
| Treatment SmallRar | *Lotus corniculatus* | True proportion | Microscopy | 0.003668 |
| Treatment SmallRar | *Leontodon hispidus* | Metabarcoding | Flow cytometry | 0.002176 |
| Treatment SmallRar | *Leontodon hispidus* | Microscopy | Flow cytometry | 0.967421 |
| Treatment SmallRar | *Leontodon hispidus* | True proportion | Flow cytometry | 0.000383 |
| Treatment SmallRar | *Leontodon hispidus* | Metabarcoding | Metabarcoding | NA |
| Treatment SmallRar | *Leontodon hispidus* | Microscopy | Metabarcoding | 0.031929 |
| Treatment SmallRar | *Leontodon hispidus* | True proportion | Metabarcoding | 0.000383 |
| Treatment SmallRar | *Leontodon hispidus* | Metabarcoding | Microscopy | NA |
| Treatment SmallRar | *Leontodon hispidus* | Microscopy | Microscopy | NA |
| Treatment SmallRar | *Leontodon hispidus* | True proportion | Microscopy | 0.000383 |
| Treatment SmallRar | *Lathyrus pratensis* | Metabarcoding | Flow cytometry | 6.50E-05 |
| Treatment SmallRar | *Lathyrus pratensis* | Microscopy | Flow cytometry | 0.406525 |
| Treatment SmallRar | *Lathyrus pratensis* | True proportion | Flow cytometry | 0.000319 |
| Treatment SmallRar | *Lathyrus pratensis* | Metabarcoding | Metabarcoding | NA |
| Treatment SmallRar | *Lathyrus pratensis* | Microscopy | Metabarcoding | 0.038315 |
| Treatment SmallRar | *Lathyrus pratensis* | True proportion | Metabarcoding | 0.000319 |
| Treatment SmallRar | *Lathyrus pratensis* | Metabarcoding | Microscopy | NA |
| Treatment SmallRar | *Lathyrus pratensis* | Microscopy | Microscopy | NA |
| Treatment SmallRar | *Lathyrus pratensis* | True proportion | Microscopy | 0.225713 |
| Treatment SmallRar | *Potentilla erecta* | Metabarcoding | Flow cytometry | 0.630529 |
| Treatment SmallRar | *Potentilla erecta* | Microscopy | Flow cytometry | 0.083916 |
| Treatment SmallRar | *Potentilla erecta* | True proportion | Flow cytometry | 0.000383 |
| Treatment SmallRar | *Potentilla erecta* | Metabarcoding | Metabarcoding | NA |
| Treatment SmallRar | *Potentilla erecta* | Microscopy | Metabarcoding | 0.067433 |
| Treatment SmallRar | *Potentilla erecta* | True proportion | Metabarcoding | 0.000383 |
| Treatment SmallRar | *Potentilla erecta* | Metabarcoding | Microscopy | NA |
| Treatment SmallRar | *Potentilla erecta* | Microscopy | Microscopy | NA |
| Treatment SmallRar | *Potentilla erecta* | True proportion | Microscopy | 0.000945 |
| Treatment SmallRar | *Prunella grandiflora* | Metabarcoding | Flow cytometry | 6.50E-05 |
| Treatment SmallRar | *Prunella grandiflora* | Microscopy | Flow cytometry | 0.000255 |
| Treatment SmallRar | *Prunella grandiflora* | True proportion | Flow cytometry | 0.000255 |
| Treatment SmallRar | *Prunella grandiflora* | Metabarcoding | Metabarcoding | NA |
| Treatment SmallRar | *Prunella grandiflora* | Microscopy | Metabarcoding | 0.061946 |
| Treatment SmallRar | *Prunella grandiflora* | True proportion | Metabarcoding | 0.000255 |
| Treatment SmallRar | *Prunella grandiflora* | Metabarcoding | Microscopy | NA |
| Treatment SmallRar | *Prunella grandiflora* | Microscopy | Microscopy | NA |
| Treatment SmallRar | *Prunella grandiflora* | True proportion | Microscopy | 0.000242 |
| Treatment SmallRar | *Plantago lanceolata* | Metabarcoding | Flow cytometry | 6.50E-05 |
| Treatment SmallRar | *Plantago lanceolata* | Microscopy | Flow cytometry | 0.000974 |
| Treatment SmallRar | *Plantago lanceolata* | True proportion | Flow cytometry | 0.000255 |
| Treatment SmallRar | *Plantago lanceolata* | Metabarcoding | Metabarcoding | NA |
| Treatment SmallRar | *Plantago lanceolata* | Microscopy | Metabarcoding | 6.50E-05 |
| Treatment SmallRar | *Plantago lanceolata* | True proportion | Metabarcoding | 0.000255 |
| Treatment SmallRar | *Plantago lanceolata* | Metabarcoding | Microscopy | NA |
| Treatment SmallRar | *Plantago lanceolata* | Microscopy | Microscopy | NA |
| Treatment SmallRar | *Plantago lanceolata* | True proportion | Microscopy | 0.115258 |
| Treatment SmallRar | *Stachys officinalis* | Metabarcoding | Flow cytometry | 6.50E-05 |
| Treatment SmallRar | *Stachys officinalis* | Microscopy | Flow cytometry | 0.105122 |
| Treatment SmallRar | *Stachys officinalis* | True proportion | Flow cytometry | 0.000255 |
| Treatment SmallRar | *Stachys officinalis* | Metabarcoding | Metabarcoding | NA |
| Treatment SmallRar | *Stachys officinalis* | Microscopy | Metabarcoding | 0.000108 |
| Treatment SmallRar | *Stachys officinalis* | True proportion | Metabarcoding | 0.000255 |
| Treatment SmallRar | *Stachys officinalis* | Metabarcoding | Microscopy | NA |
| Treatment SmallRar | *Stachys officinalis* | Microscopy | Microscopy | NA |
| Treatment SmallRar | *Stachys officinalis* | True proportion | Microscopy | 0.002842 |

Supplementary figure S10. Comparison of relative proportions of pollen plant families among all three methods (Microscopy, MIFC and metabarcoding) estimated for 30 pollinators collected in the field with “blind” analysis.


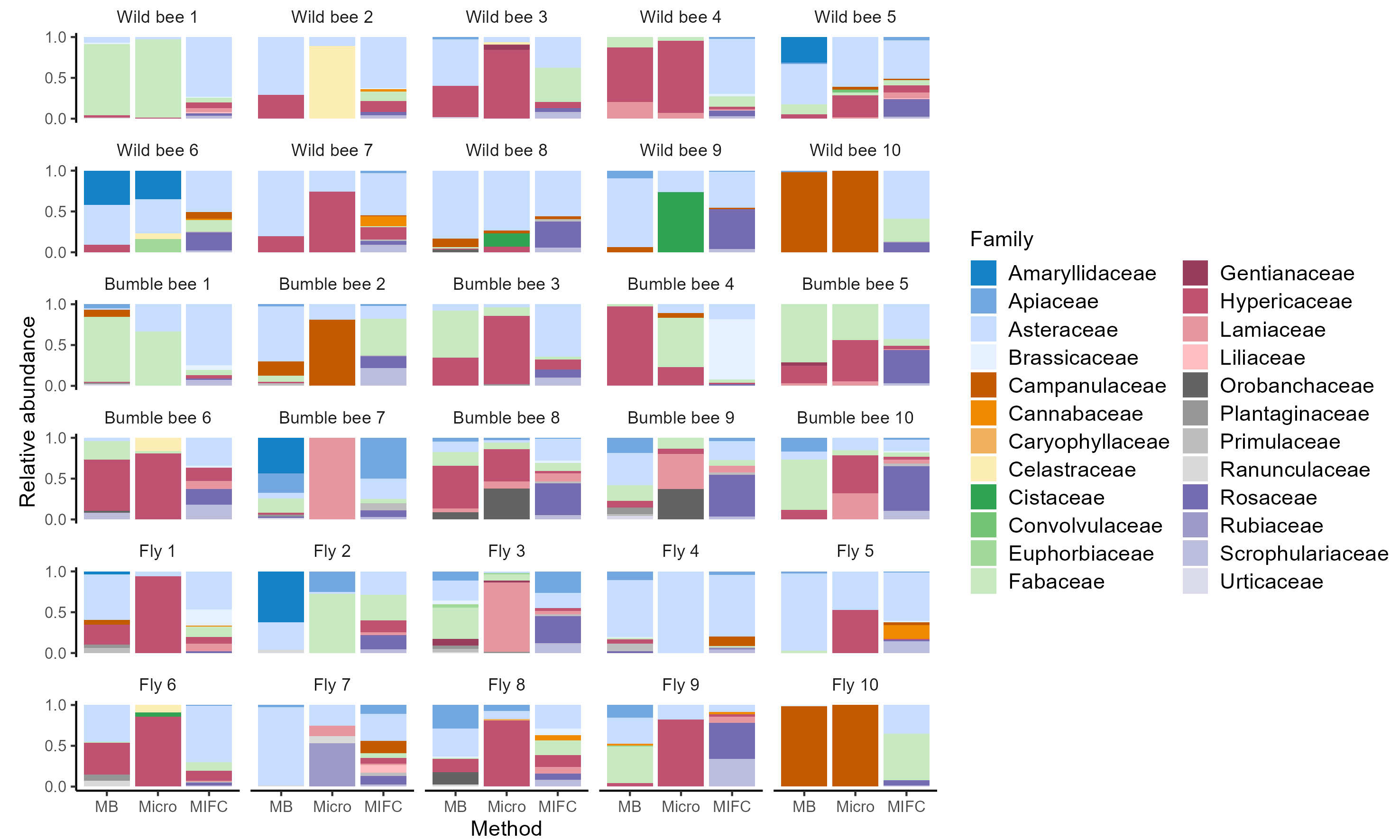


Supplemental figure S11. Linear regression lines show the variability of the identification results of each pairwise method comparison for the “blind” analysis for a) Metabarcoding-Microscopy, b) Metabarcoding-MIFC, and c) MIFC-Microscopy.


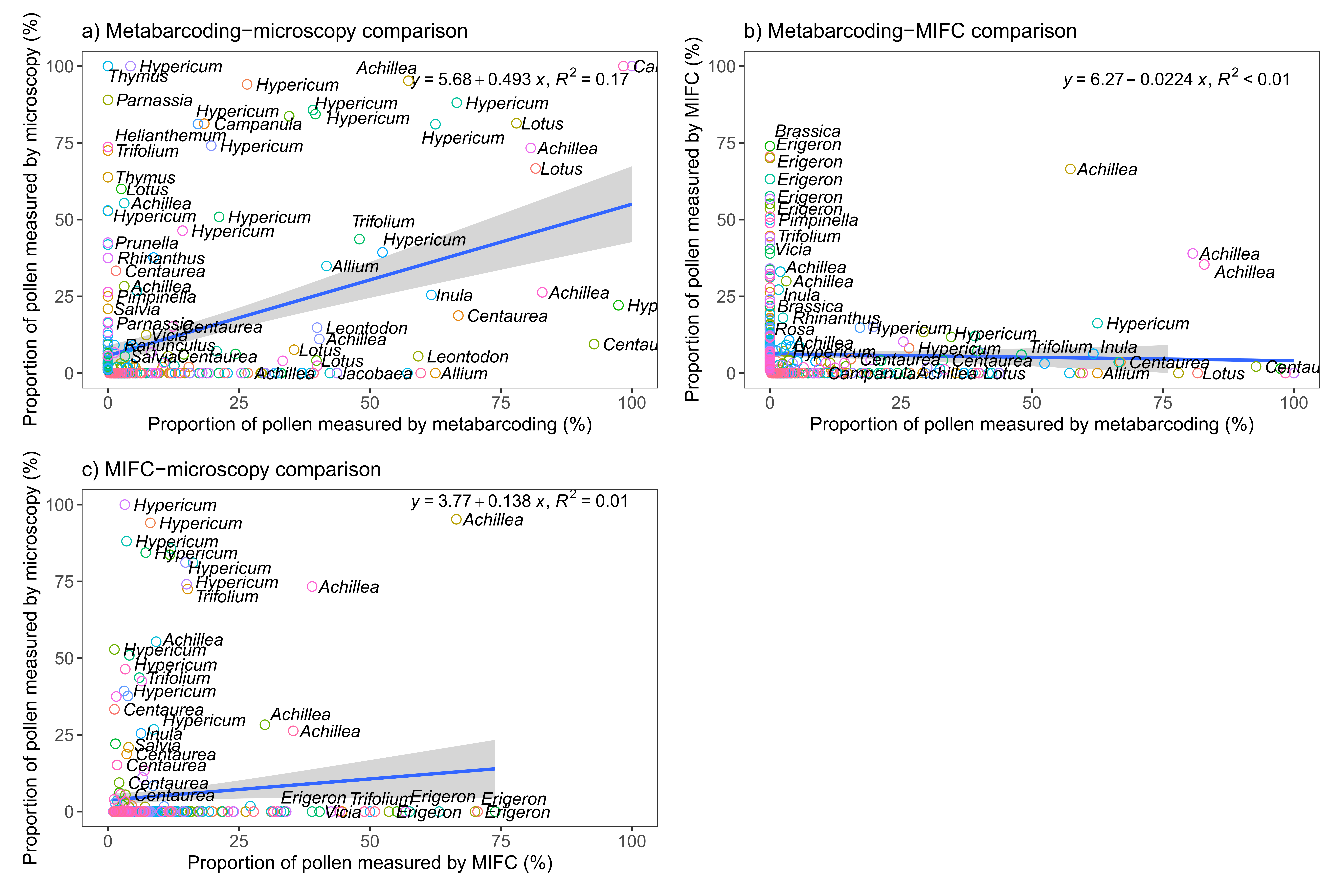


Supplemental figure S12. Linear regression lines show the variability of the identification results of each pairwise method comparison for the “informed” analysis for a) Metabarcoding-Microscopy, b) Metabarcoding-MIFC, and c) MIFC-Microscopy.


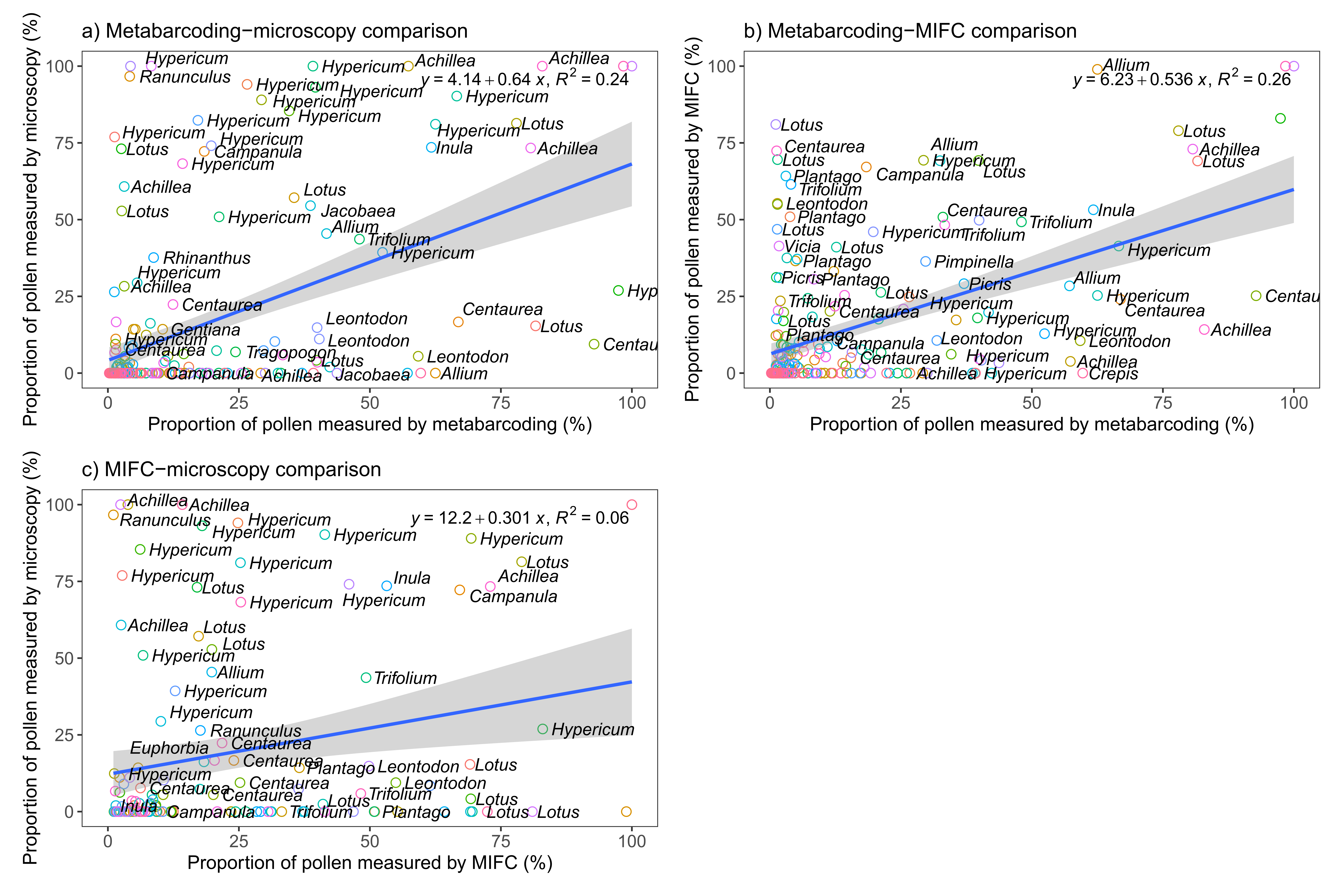


Supplementary table S13. A summary of the detection results of the positive and negative controls implemented in the artificial mixtures and the insect pollen with each method.

| **Control type** | **Control** | **Method** | **Genus Composition** | **Number of pollen grains/ ITS2 reads (MB)** | **Proportion** |
| --- | --- | --- | --- | --- | --- |
| **Artificial Mixture** | | | | | |
| Positive control | *Centaurea phrygia* | Microscopy | Centaurea | 28 | 1 |
| Positive control | *Hypericum perforatum* | Microscopy | Hypericum | 25 | 1 |
| Positive control | *Lathyrus pratensis* | Microscopy | Campanula | 1 | 0.01538462 |
| Positive control | *Lathyrus pratensis* | Microscopy | Crepis | 1 | 0.01538462 |
| Positive control | *Lathyrus pratensis* | Microscopy | Hypericum | 2 | 0.03076923 |
| Positive control | *Lathyrus pratensis* | Microscopy | Lotus | 3 | 0.04615385 |
| Positive control | *Lathyrus pratensis* | Microscopy | Vicia | 58 | 0.89230769 |
| Positive control | *Leontodon hispidus* | Microscopy | Campanula | 1 | 0.03571429 |
| Positive control | *Leontodon hispidus* | Microscopy | Crepis | 9 | 0.32142857 |
| Positive control | *Leontodon hispidus* | Microscopy | Hypericum | 18 | 0.64285714 |
| Positive control | *Lotus corniculatus* | Microscopy | Lotus | 31 | 1 |
| Positive control | *Plantago lanceolata* | Microscopy | Plantago | 31 | 1 |
| Positive control | *Potentilla erecta* | Microscopy | Potentilla | 22 | 1 |
| Positive control | *Prunella grandiflora* | Microscopy | Campanula | 1 | 0.01587302 |
| Positive control | *Prunella grandiflora* | Microscopy | Hypericum | 1 | 0.01587302 |
| Positive control | *Prunella grandiflora* | Microscopy | Parnassia | 3 | 0.04761905 |
| Positive control | *Prunella grandiflora* | Microscopy | Prunella | 24 | 0.38095238 |
| Positive control | *Prunella grandiflora* | Microscopy | Stachys | 1 | 0.01587302 |
| Positive control | *Prunella grandiflora* | Microscopy | Thymus | 33 | 0.52380952 |
| Positive control | *Stachys officinalis* | Microscopy | Stachys | 28 | 1 |
| Positive control | *Centaurea phrygia* | Metabarcoding | Centaurea | 17400 | 0.79757976 |
| Positive control | *Centaurea phrygia* | Metabarcoding | Knautia | 1090 | 0.04996333 |
| Positive control | *Centaurea phrygia* | Metabarcoding | Lupinus | 3326 | 0.15245691 |
| Positive control | *Hypericum perforatum* | Metabarcoding | Calluna | 1040 | 0.02233821 |
| Positive control | *Hypericum perforatum* | Metabarcoding | Hypericum | 41351 | 0.88818008 |
| Positive control | *Hypericum perforatum* | Metabarcoding | Knautia | 4166 | 0.08948171 |
| Positive control | *Lathyrus pratensis* | Metabarcoding | Crepis | 256 | 0.01112173 |
| Positive control | *Lathyrus pratensis* | Metabarcoding | Hypericum | 342 | 0.01485794 |
| Positive control | *Lathyrus pratensis* | Metabarcoding | Knautia | 818 | 0.03553741 |
| Positive control | *Lathyrus pratensis* | Metabarcoding | Lathyrus | 17038 | 0.74020332 |
| Positive control | *Lathyrus pratensis* | Metabarcoding | Leontodon | 501 | 0.02176557 |
| Positive control | *Lathyrus pratensis* | Metabarcoding | Lotus | 3618 | 0.15718134 |
| Positive control | *Lathyrus pratensis* | Metabarcoding | Stachys | 445 | 0.0193327 |
| Positive control | *Leontodon hispidus* | Metabarcoding | Centaurea | 1102 | 0.0340134 |
| Positive control | *Leontodon hispidus* | Metabarcoding | Crepis | 1310 | 0.04043335 |
| Positive control | *Leontodon hispidus* | Metabarcoding | Hieracium | 352 | 0.01086453 |
| Positive control | *Leontodon hispidus* | Metabarcoding | Hypericum | 5216 | 0.16099262 |
| Positive control | *Leontodon hispidus* | Metabarcoding | Knautia | 978 | 0.03018612 |
| Positive control | *Leontodon hispidus* | Metabarcoding | Leontodon | 19998 | 0.61724127 |
| Positive control | *Leontodon hispidus* | Metabarcoding | Leucanthemum | 594 | 0.0183339 |
| Positive control | *Leontodon hispidus* | Metabarcoding | Lotus | 542 | 0.01672891 |
| Positive control | *Leontodon hispidus* | Metabarcoding | Peucedanum | 558 | 0.01722275 |
| Positive control | *Leontodon hispidus* | Metabarcoding | Potentilla | 674 | 0.02080311 |
| Positive control | *Leontodon hispidus* | Metabarcoding | Trifolium | 1075 | 0.03318004 |
| Positive control | *Lotus corniculatus* | Metabarcoding | Calluna | 719 | 0.01112504 |
| Positive control | *Lotus corniculatus* | Metabarcoding | Knautia | 8167 | 0.12636742 |
| Positive control | *Lotus corniculatus* | Metabarcoding | Lotus | 54743 | 0.84703461 |
| Positive control | *Lotus corniculatus* | Metabarcoding | Plantago | 1000 | 0.01547293 |
| Positive control | *Plantago lanceolata* | Metabarcoding | Plantago | 75591 | 1 |
| Positive control | *Potentilla erecta* | Metabarcoding | Calluna | 1640 | 0.02086833 |
| Positive control | *Potentilla erecta* | Metabarcoding | Knautia | 1060 | 0.01348806 |
| Positive control | *Potentilla erecta* | Metabarcoding | Potentilla | 75888 | 0.96564361 |
| Positive control | *Prunella grandiflora* | Metabarcoding | Centaurea | 366 | 0.01634366 |
| Positive control | *Prunella grandiflora* | Metabarcoding | Hypericum | 312 | 0.0139323 |
| Positive control | *Prunella grandiflora* | Metabarcoding | Knautia | 1973 | 0.08810396 |
| Positive control | *Prunella grandiflora* | Metabarcoding | Potentilla | 380 | 0.01696883 |
| Positive control | *Prunella grandiflora* | Metabarcoding | Prunella | 11008 | 0.49156024 |
| Positive control | *Prunella grandiflora* | Metabarcoding | Stachys | 482 | 0.02152362 |
| Positive control | *Prunella grandiflora* | Metabarcoding | Trifolium | 7873 | 0.35156738 |
| Positive control | *Stachys officinalis* | Metabarcoding | Hypericum | 391 | 0.01400229 |
| Positive control | *Stachys officinalis* | Metabarcoding | Knautia | 836 | 0.0299384 |
| Positive control | *Stachys officinalis* | Metabarcoding | Lamiaceae spc | 309 | 0.01106575 |
| Positive control | *Stachys officinalis* | Metabarcoding | Stachys | 26388 | 0.94499355 |
| Positive control | *Centaurea phrygia* | MIFC | Centaurea | 431 | 0.93492408 |
| Positive control | *Centaurea phrygia* | MIFC | Plantago | 11 | 0.02386117 |
| Positive control | *Centaurea phrygia* | MIFC | Rosa | 14 | 0.03036876 |
| Positive control | *Centaurea phrygia* | MIFC | Trifolium | 5 | 0.01084599 |
| Positive control | *Hypericum perforatum* | MIFC | Hypericum | 311 | 0.88101983 |
| Positive control | *Hypericum perforatum* | MIFC | Lotus | 29 | 0.08215297 |
| Positive control | *Hypericum perforatum* | MIFC | Torilis | 13 | 0.0368272 |
| Positive control | *Lathyrus pratensis* | MIFC | Campanula | 10 | 0.24390244 |
| Positive control | *Lathyrus pratensis* | MIFC | Hypericum | 4 | 0.09756098 |
| Positive control | *Lathyrus pratensis* | MIFC | Lathyrus | 15 | 0.36585366 |
| Positive control | *Lathyrus pratensis* | MIFC | Lotus | 4 | 0.09756098 |
| Positive control | *Lathyrus pratensis* | MIFC | Plantago | 3 | 0.07317073 |
| Positive control | *Lathyrus pratensis* | MIFC | Potentilla | 1 | 0.02439024 |
| Positive control | *Lathyrus pratensis* | MIFC | Rosa | 1 | 0.02439024 |
| Positive control | *Lathyrus pratensis* | MIFC | Trifolium | 1 | 0.02439024 |
| Positive control | *Lathyrus pratensis* | MIFC | Vicia | 2 | 0.04878049 |
| Positive control | *Leontodon hispidus* | MIFC | Achillea | 30 | 0.11627907 |
| Positive control | *Leontodon hispidus* | MIFC | Centaurea | 4 | 0.01550388 |
| Positive control | *Leontodon hispidus* | MIFC | Cichorium | 24 | 0.09302326 |
| Positive control | *Leontodon hispidus* | MIFC | Leontodon | 89 | 0.34496124 |
| Positive control | *Leontodon hispidus* | MIFC | Picris | 75 | 0.29069767 |
| Positive control | *Leontodon hispidus* | MIFC | Plantago | 4 | 0.01550388 |
| Positive control | *Leontodon hispidus* | MIFC | Rhinanthus | 9 | 0.03488372 |
| Positive control | *Leontodon hispidus* | MIFC | Rosa | 9 | 0.03488372 |
| Positive control | *Leontodon hispidus* | MIFC | Salvia | 3 | 0.01162791 |
| Positive control | *Leontodon hispidus* | MIFC | Torilis | 4 | 0.01550388 |
| Positive control | *Leontodon hispidus* | MIFC | Trifolium | 7 | 0.02713178 |
| Positive control | *Lotus corniculatus* | MIFC | Lotus | 95 | 0.95 |
| Positive control | *Lotus corniculatus* | MIFC | Pimpinella | 5 | 0.05 |
| Positive control | *Plantago lanceolata* | MIFC | Artemisia | 4 | 0.01257862 |
| Positive control | *Plantago lanceolata* | MIFC | Campanula | 33 | 0.10377358 |
| Positive control | *Plantago lanceolata* | MIFC | Hypericum | 8 | 0.02515723 |
| Positive control | *Plantago lanceolata* | MIFC | Pimpinella | 4 | 0.01257862 |
| Positive control | *Plantago lanceolata* | MIFC | Plantago | 242 | 0.76100629 |
| Positive control | *Plantago lanceolata* | MIFC | Prunella | 8 | 0.02515723 |
| Positive control | *Plantago lanceolata* | MIFC | Trifolium | 7 | 0.02201258 |
| Positive control | *Plantago lanceolata* | MIFC | Verbascum | 12 | 0.03773585 |
| Positive control | *Potentilla erecta* | MIFC | Campanula | 4 | 0.025 |
| Positive control | *Potentilla erecta* | MIFC | Hypericum | 3 | 0.01875 |
| Positive control | *Potentilla erecta* | MIFC | Lotus | 3 | 0.01875 |
| Positive control | *Potentilla erecta* | MIFC | Pimpinella | 9 | 0.05625 |
| Positive control | *Potentilla erecta* | MIFC | Plantago | 35 | 0.21875 |
| Positive control | *Potentilla erecta* | MIFC | Potentilla | 97 | 0.60625 |
| Positive control | *Potentilla erecta* | MIFC | Torilis | 3 | 0.01875 |
| Positive control | *Potentilla erecta* | MIFC | Verbascum | 6 | 0.0375 |
| Positive control | *Prunella grandiflora* | MIFC | Campanula | 7 | 0.02201258 |
| Positive control | *Prunella grandiflora* | MIFC | Centaurea | 4 | 0.01257862 |
| Positive control | *Prunella grandiflora* | MIFC | Plantago | 10 | 0.03144654 |
| Positive control | *Prunella grandiflora* | MIFC | Prunella | 157 | 0.49371069 |
| Positive control | *Prunella grandiflora* | MIFC | Rhinanthus | 6 | 0.01886792 |
| Positive control | *Prunella grandiflora* | MIFC | Rosa | 12 | 0.03773585 |
| Positive control | *Prunella grandiflora* | MIFC | Salvia | 22 | 0.06918239 |
| Positive control | *Prunella grandiflora* | MIFC | Stachys | 4 | 0.01257862 |
| Positive control | *Prunella grandiflora* | MIFC | Trifolium | 96 | 0.30188679 |
| Positive control | *Stachys officinalis* | MIFC | Campanula | 4 | 0.4 |
| Positive control | *Stachys officinalis* | MIFC | Hypericum | 2 | 0.2 |
| Positive control | *Stachys officinalis* | MIFC | Plantago | 2 | 0.2 |
| Positive control | *Stachys officinalis* | MIFC | Stachys | 2 | 0.2 |
| Negative control | NC1 | Microscopy | *-* | 0 | 0 |
|  | NC2 |  | *-* | 0 | 0 |
| Negative control | NC1 | Metabarcoding | Calluna | 3319 | 0.06915016 |
| Negative control | NC1 | Metabarcoding | Centaurea | 3715 | 0.07740067 |
| Negative control | NC1 | Metabarcoding | Knautia | 13636 | 0.28410109 |
| Negative control | NC1 | Metabarcoding | Leymus | 1335 | 0.02781424 |
| Negative control | NC1 | Metabarcoding | Lupinus | 3772 | 0.07858825 |
| Negative control | NC1 | Metabarcoding | Secale | 19746 | 0.41140071 |
| Negative control | NC1 | Metabarcoding | Solanum | 2474 | 0.05154489 |
| Negative control | NC2 | Metabarcoding | Brassica | 421 | 0.01321904 |
| Negative control | NC2 | Metabarcoding | Calluna | 2901 | 0.09108892 |
| Negative control | NC2 | Metabarcoding | Centaurea | 439 | 0.01378423 |
| Negative control | NC2 | Metabarcoding | Dianthus | 923 | 0.02898141 |
| Negative control | NC2 | Metabarcoding | Knautia | 21726 | 0.68217784 |
| Negative control | NC2 | Metabarcoding | Lupinus | 4228 | 0.13275559 |
| Negative control | NC2 | Metabarcoding | Plantago | 738 | 0.02317257 |
| Negative control | NC2 | Metabarcoding | Scabiosa | 472 | 0.0148204 |
| Negative control | NC1 | MIFC | *-* | 0 | 0 |
|  | NC2 |  | *-* | 0 | 0 |
| **Insect pollen** | | | | | |
| Negative control | NC1 | Microscopy | - | 0 | 0 |
|  | NC2 |  | - | 0 | 0 |
| Negative control | NC1 | Metabarcoding | Barbarea | 11 | 0.01492537 |
| Negative control | NC1 | Metabarcoding | Betula | 35 | 0.04748982 |
| Negative control | NC1 | Metabarcoding | Brassica | 4 | 0.00542741 |
| Negative control | NC1 | Metabarcoding | Calluna | 20 | 0.02713704 |
| Negative control | NC1 | Metabarcoding | Campanula | 4 | 0.00542741 |
| Negative control | NC1 | Metabarcoding | Centaurea | 8 | 0.01085482 |
| Negative control | NC1 | Metabarcoding | Crepis | 440 | 0.59701493 |
| Negative control | NC1 | Metabarcoding | Hedera | 100 | 0.13568521 |
| Negative control | NC1 | Metabarcoding | Hypericum | 27 | 0.03663501 |
| Negative control | NC1 | Metabarcoding | Inula | 10 | 0.01356852 |
| Negative control | NC1 | Metabarcoding | Lotus | 13 | 0.01763908 |
| Negative control | NC1 | Metabarcoding | Picris | 16 | 0.02170963 |
| Negative control | NC1 | Metabarcoding | Plantago | 2 | 0.0027137 |
| Negative control | NC1 | Metabarcoding | Sambucus | 47 | 0.06377205 |
| Negative control | NC2 | Metabarcoding | Acer | 213 | 0.39226519 |
| Negative control | NC2 | Metabarcoding | Allium | 4 | 0.00736648 |
| Negative control | NC2 | Metabarcoding | Brassica | 65 | 0.11970534 |
| Negative control | NC2 | Metabarcoding | Calluna | 33 | 0.06077348 |
| Negative control | NC2 | Metabarcoding | Capsella | 6 | 0.01104972 |
| Negative control | NC2 | Metabarcoding | Circaea | 4 | 0.00736648 |
| Negative control | NC2 | Metabarcoding | Cirsium | 4 | 0.00736648 |
| Negative control | NC2 | Metabarcoding | Crepis | 121 | 0.2228361 |
| Negative control | NC2 | Metabarcoding | Hedera | 26 | 0.04788214 |
| Negative control | NC2 | Metabarcoding | Picris | 5 | 0.0092081 |
| Negative control | NC2 | Metabarcoding | Potentilla | 2 | 0.00368324 |
| Negative control | NC2 | Metabarcoding | Ranunculus | 7 | 0.01289134 |
| Negative control | NC2 | Metabarcoding | Rosa | 35 | 0.06445672 |
| Negative control | NC2 | Metabarcoding | Trifolium | 14 | 0.02578269 |
| Negative control | NC2 | Metabarcoding | Urtica | 4 | 0.00736648 |
| Negative control | NC1 | MIFC | - | 0 | 0 |
|  | NC2 |  | - | 0 | 0 |

Supplemental table S14. Representative pollen reference images for each artificial mixture species compared to the representative images of the analysed samples.

|  | Reference images | Artificial mixture images |
| --- | --- | --- |
| *Lotus corniculatus* | 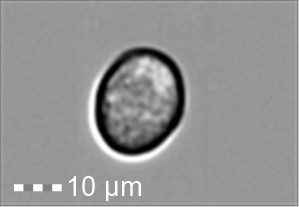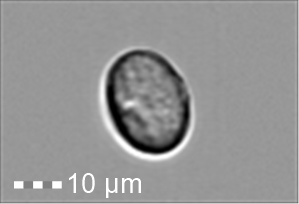 | 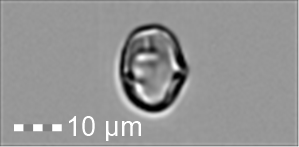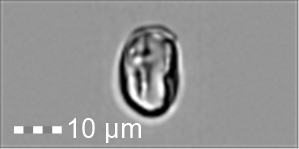 |
| *Leoontodon hispidus* | 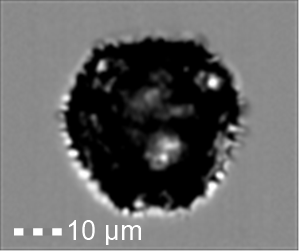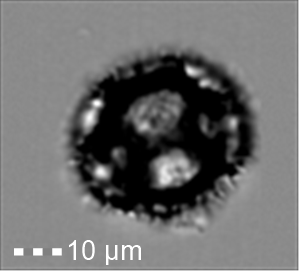 | 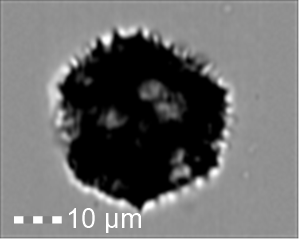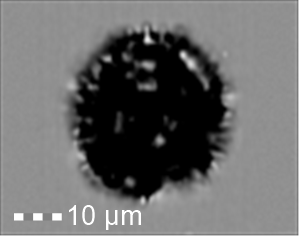 |
| *Lathyrus pratensis* | 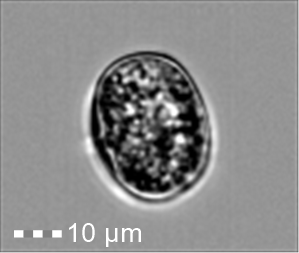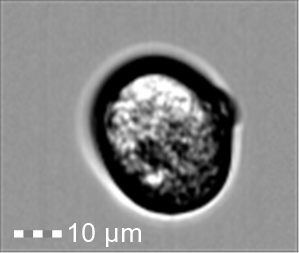 | 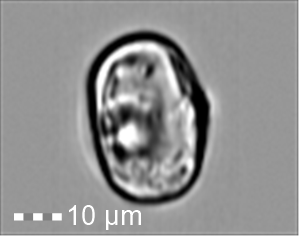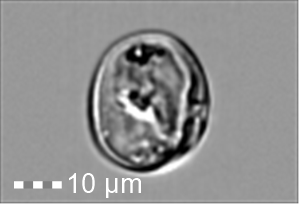 |
| *Potentilla erecta* | 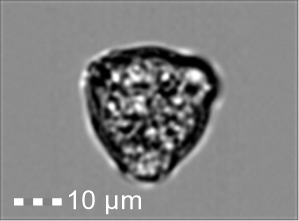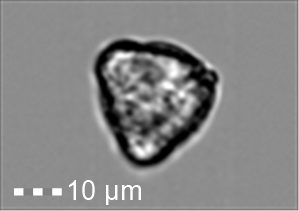 | 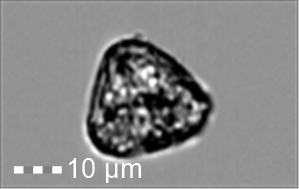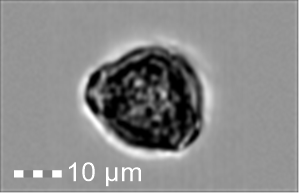 |
| *Prunella vulgaris* | 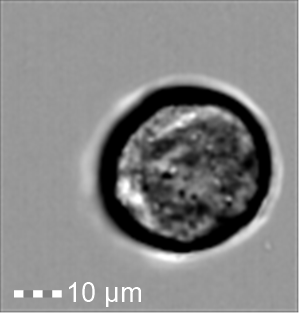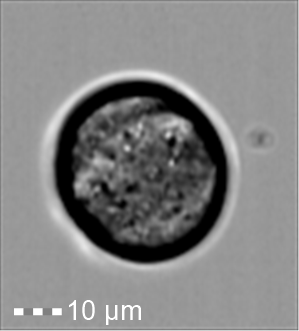 | 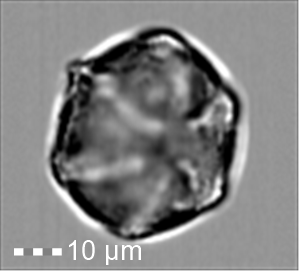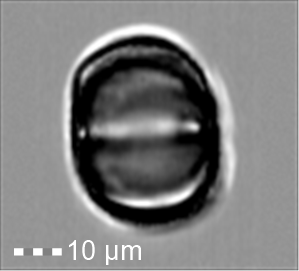 |
| *Plantago lanceolata* | 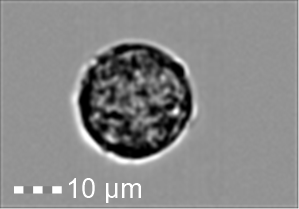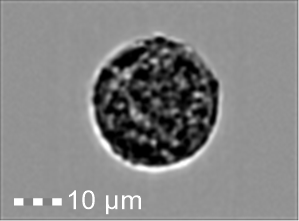 | 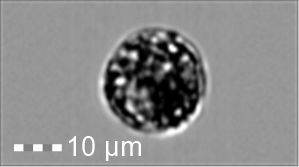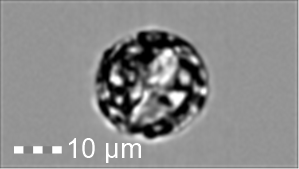 |
| *Hypericum perforatum* | 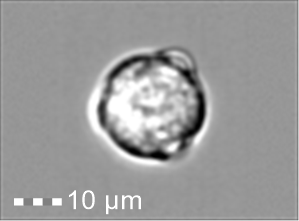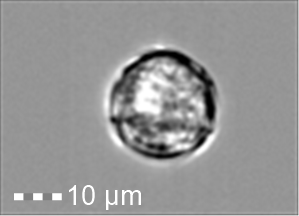 | 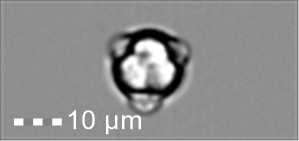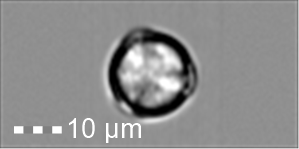 |
| *Centaurea phrygia* | 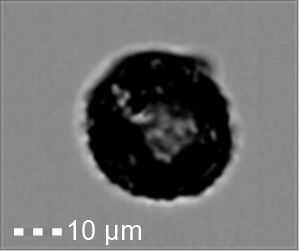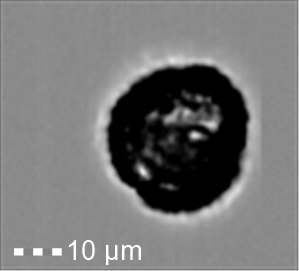 | 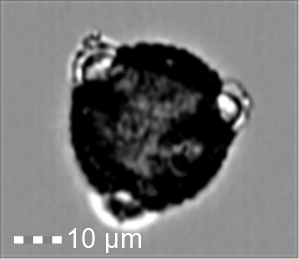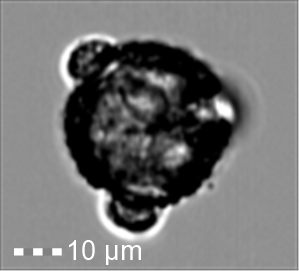 |
| *Stachys officinalis* | 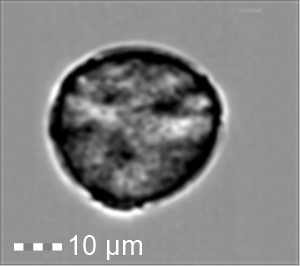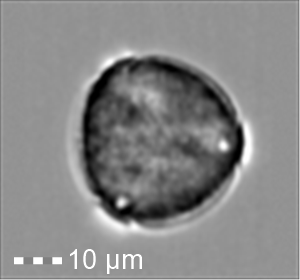 | 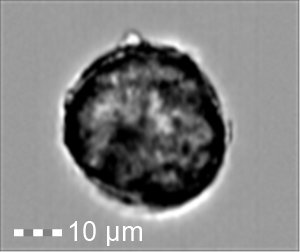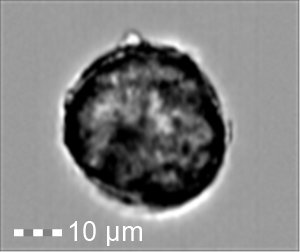 |
